# Dietary Tannic Acid Improves Hepatic Health and Capacity to Deal with Temperature Fluctuation in the Chinese Soft-Shelled Turtle (*Pelodiscus sinensis*)

**DOI:** 10.3390/ani15040544

**Published:** 2025-02-13

**Authors:** Liqin Ji, Yisen Shangguan, Qing Shi, Zhen Dong, Chen Chen, Junxian Zhu, Xiaoyou Hong, Xiaoli Liu, Chengqing Wei, Xinping Zhu, Wei Li

**Affiliations:** 1 Key Laboratory of Tropical and Subtropical Fishery Resources Application and Cultivation, Ministry of Agriculture and Rural Affairs, Pearl River Fisheries Research Institute, Chinese Academy of Fishery Sciences, Guangzhou 510275, China; goofyji@126.com (L.J.); sgysss1022@163.com (Y.S.);; 2 Key Laboratory of Marine Environmental Survey Technology and Application, South China Sea Marine Survey Center, Ministry of Natural Resources, Guangzhou 510275, China

**Keywords:** Chinese soft-shelled turtle, liver, tannin acid, histology, metabolites, signaling pathways

## Abstract

To assess the impact of tannic acid (TA) on the hepatic health of the Chinese soft-shelled turtle, the individuals were fed diets containing 0 (CG), 0.5, 1, 2, and 4 g/kg TA diets for 60 days (0 hps). Afterwards, the turtles were challenged with 15 °C cold stress for 24 h (24 hps) and then recovered to 28 °C for 24 h (48 hps). The results indicated that 4 g/kg TA has a significant toxic effect on the turtles after 60-day administration. The hepatic T-SOD, CAT, GSH-Px, and T-AOC activities in the TA2 were increased at 0 hps and 24 hps (*p* < 0.05) compared with CG. Similarly, *Sod1*, *Sod2*, *Cat*, *Gsh-px3*, and *Gsh-px4* mRNA levels in the TA2 were higher than in the other four groups at 0 hps and 24 hps (*p* < 0.05). The histology data showed that 4 g/kg TA induced injuries in liver at 0 hps and after temperature fluctuation, whereas the 2 g/kg TA effectively attenuated the hepatic injuries. A total of 202 differentially expressed metabolites (DEMs) in the CG vs. TA2 and 115 DEMs in the LTCG vs. LTTA2 were separately detected by the metabolome. The cephalosporin C, protoporphyrin, bis(2-ethylhexyl) phthalate, 2-acetamidoethylphosphonat, verbasosid, and norcocain, were obvious DEMs in the CG vs. TA2. Furthermore, valienone 7-phosphate, 5-HETE, pregnanetriolone, 4-keto-anhydrotetracyclin, dephospho-CoA, and lysoPC(18:4(6Z,9Z,12Z,15Z)/0:0) were top DEMs in the LTCG vs. LTTA2. The “adipocytokine signaling pathway” and “AMPK signaling pathway” were the key pathways enriched in the CG vs. TA2, while “fatty acid biosynthesis”, “steroid biosynthesis”, and “linoleic acid metabolism” were most abundant in the LTCG vs. LTTA2. Generally, this research indicated that 2 g/kg TA could protect hepatic health from temperature fluctuations by improving antioxidant capacity, reducing histological injuries, and regulating lipid-related signaling pathways.

## Simple Summary

Great temperature variations increase the risk of illness or death in the Chinese soft-shelled turtle, potentially leading to significant economic losses in the farming industry. Therefore, it is crucial to find effective strategies to overcome temperature stress-induced damage in the farming industry. Herein, we evaluated the roles of dietary tannic acid in regulating liver health under temperature fluctuations. The results found that 2 g/kg dietary tannic acid improved the activities of antioxidant enzymes and alleviated histological injuries in the liver. Furthermore, it affected the metabolic profiles and regulated energy-related signaling pathways in the liver to deal with cold stress, such as adipocytokine signaling pathway, steroid biosynthesis, and α-linolenic acid metabolism. Generally, this research indicated that 2 g/kg TA could protect hepatic health from temperature fluctuations by enhancing the antioxidant capacity, reducing histological injuries, and regulating lipid-related signaling pathways.

## 1. Introduction

The Chinese soft-shelled turtle (*Pelodiscus sinensis*) is popular as an economical aquaculture species in China, Japan, and other Asian countries due to its rich nutrients and exceptional medicinal qualities [1]. In China, the Chinese soft-shelled turtle is extensively cultivated owing to the growing demand in the market, with an annual harvest of close to 0.5 million tons in 2023 [2]. To effectively improve yield and decline land occupation, farming modes have tended to change from extensive farming to intensive farming. However, high stocking density makes the animals more vulnerable and sensitive when they are exposed to adverse factors [3]. As a type of warm-water reptile, the Chinese soft-shelled turtle faces massive death rates when facing sharp temperature fluctuations within the farming industry [4]. For example, the temperature gap between day and night can reach up to 20 °C during the spring and autumn in extreme cases [5]. This great temperature variation increases the risk of illness or death in farmed turtles, potentially leading to significant economic losses [6]. Therefore, it is urgent to explore effective and safe strategies to protect the Chinese soft-shelled turtle from being damaged by temperature fluctuation, which will promote the sustainable development of the Chinese soft-shelled turtle farming industry.

To enhance immunity and stress resistance in aquaculture, the World Health Organization (WHO) and the Food and Agriculture Organization of the United Nations (FAO) promote natural immunostimulants for prophylactic use, owing to their safety and easy availability [7]. Of the natural immunostimulants, numerous plant extracts have attracted attention as potential feed additives, attributed to their good performance in enhancing immunity and optimizing the physiological status of cultured animals [8]. Tannic acid (TA), with the chemical formula C_76_H_52_O_46_, is composed of 10 molecules of gallic acid attached to a central glucose unit [9]. As one of the natural botanical additives, TA can be extracted from various plants, such as chestnuts, tea, red grape, and spruce [10]. It has been reported that adding TAs to diets can promote animal growth and the capacity to resist stimuli. For instance, Yang et al. suggest that supplementing the diet with 3.75 g/kg TA can improve the growth performance and feed utilization in largemouth bass [11]. Yang et al. have found that TA inhibits BPA-induced pyroptosis of hepatocytes via the PTEN/P13K/AKT pathway in grass carp [12]. In addition, TA is applied as a biological antioxidant for broiler chickens to cope with heat stress [13]. In broilers co-infected with Coccidia and clostidium perfringens, 1000 mg/kg dietary TA increased the hepatic antioxidant capacity by activating the transcription factor Nrf2 downstream of the Nrf2-Keap1 pathway [14].

The liver is a critical organ in the organism responsible for a series of functions, including metabolism, immunity, digestion, detoxification, etc. [15]. The liver has a dual blood supply from the portal vein (approximately 75%) and the hepatic artery (approximately 25%). The unique dual blood supply can provide nutritional metabolism and clear toxins from the splanchnic area [16]. The liver is both a site of anabolism and catabolism, which plays an important role in the metabolism of carbohydrates, proteins, amino acids, and lipids. It has been reported that the liver can mobilize the energy metabolism to maintain homeostasis when organisms are exposed to sudden temperature fluctuations [17,18]. For example, heat stress on the American shad causes significant changes in the hepatic substances, such as in the presence of amino acids, starch, glucose, choline, and betaine. This affects important energy metabolic pathways for carbohydrates, proteins, and glucose [19]. Therefore, it is a predominant organ for researchers in discovering the variation of metabolic profiles in organisms under various stimuli. The effect of TA on liver metabolism has been studied in animals. For instance, TA can protect the liver of broilers by inhibiting hepatic apoptosis and pyroptosis via the inactivation of the TLR4/MyD88/MF-kB signaling pathway [20]. However, research on the impact of TAs on liver function under external stress is still lacking for the Chinese soft-shelled turtle. Hence, this study performed a 60-day feeding trial, followed by a low-temperature challenge, to systematically investigate the roles of dietary TA in regulating liver health under temperature fluctuations through the histology and metabolomic analysis of the liver. Our results provide a theoretical foundation for applying TA in the Chinese soft-shelled turtle farming industry.

## 2. Material and Methods

### 2.1. Experimental Diets

The TA extracted from chestnut (purity = 75%) was purchased from Pureland Co., Ltd. (Guangzhou, China). The TA contained the following impurities: starch, protein, lipid, inorganic salt, and gelatin. These components had no phenolic hydroxyl group or antioxidant function, and would not influence the effect of TA in this research. Five iso-proteic (about 462 g kg^−1^ crude protein) and iso-lipidic (about 76 g kg^−1^ crude lipid) diets containing different levels of TA (0, 0.5, 1, 2, and 4 g kg^−1^ of the diet) were separately defined as the CG, TA0.5, TA1, TA2, and TA4. The experimental diets were processed with the formulation shown in Table 1, machined into pellets by the Guangdong Nutriera Group Co., Ltd. (Guangzhou, China). In brief, all raw materials were crushed, sifted (80 mesh), and fully mixed through the blender after weighing. The dough was then extruded into soft pellets with a 1.5 mm diameter by an expansion mechanism. After drying at 55 °C for 12 h, the pellets were preserved in airtight bags at room temperature for the feeding trial.

### 2.2. Animal Management in the Feeding Trial

After obtaining the gifted Chinese soft-shelled turtles from Huizhou Wealth Xing Industrial Co., Ltd. (Huizhou, China), these individuals were acclimatized for two weeks before the feeding trial in the Pearl River Fisheries Research Institute, Chinese Academy of Fishery Sciences (Guangzhou, China). After the 14-day acclimation, 1050 healthy turtles with an average body weight of 12.54 ± 0.54 g were unbiasedly assigned to 15 polyethylene tanks (1 m × 1 m × 0.25 m) at 70 turtles per tank stocking density. The random selection of turtles was achieved by human choice without weight and appearance bias. Each group comprised three tanks as biological replicates.

Turtles were fed twice daily (9:00 and 17:00) for 60 days. An apparent satiation state was exhibited in that a small amount of the pellet diet settled in the tank, and most turtles demonstrated no feeding behaviour during each meal. An hour after feeding, residual bait and feces were removed by pipette. About one-third of the tank water was replaced every 3 days. The water temperature was stable at 28 ± 3 °C, and the other water parameters were maintained as follows: pH, 8.0 ± 0.4; dissolved oxygen, 6.0 ± 1.7 mg/L; NH_3_-N, 4.0 ± 1.2 mg/L; NO_2_^-^, 1.0 ± 0.4 mg/L; and alkalinity, 45 ± 4. The number of dead turtles was recorded daily during the feeding trial. The cumulative mortality was calculated by the following formula.

Cumulative mortality (%) = 100% × (N_d_/N_t_)

(N_t_ was the total turtle number at the beginning of the feeding trial. N_d_ was the cumulative dead turtle number during the feeding trial)

### 2.3. Temperature Fluctuating Experiment

**Figure 1:**
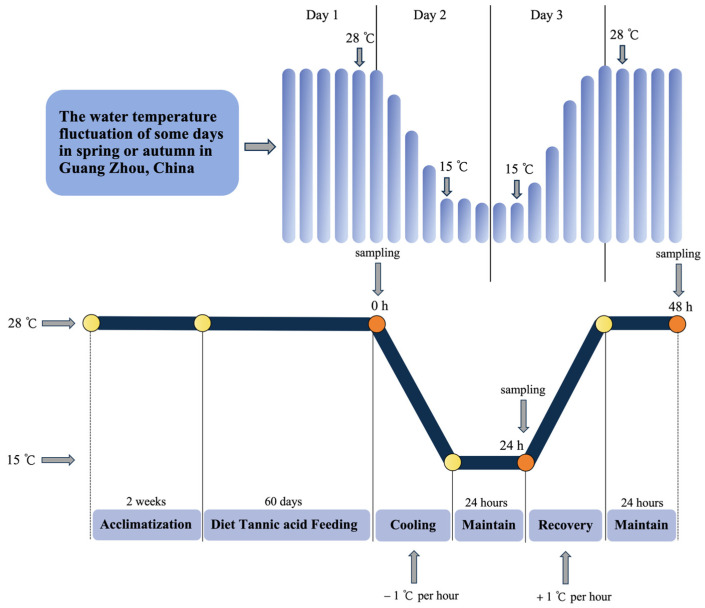
Experimental diagram.

The experiment diagram is shown in Figure 1. After 60 days of feeding, 10 turtles in each group were sampled, as the initial point of the temperature experiment, which was designated as 0 h post-cold stress (hps). Then, 30 individuals from each group were randomly assigned to 2 plastic boxes (37 cm × 25 cm × 11 cm) for the following temperature fluctuating experiment. After 24 h of starving, the turtles were transferred to RXZ-436 incubators (Ningbo Jiangnan Instrument Factory, Ningbo, China) to start the experiment. In the first phase, the water temperature decreased from 28 °C to 15 °C at the rate of −1 °C per hour and kept at 15 °C for 24 h, which was appointed as 24 hps. Then, in the second phase, the water temperature rose from 15 °C to 28 °C at the rate of 1 °C per hour and was kept at 28 °C for 24 h, which was deemed as 48 hps.

### 2.4. Sample Collection

The Chinese soft-shelled turtles in five groups were sampled at 0, 24, and 48 hps following the 60-day feeding experiment. At each time point, 6 turtles were sampled in each group (*n* = 6). The turtles were rapidly dissected to collect the liver after being anesthetized with 1 g/L 3-aminobenzoic acid ethyl ester methane-sulfonate (MS-222) solution. A small part of the liver (1 cm × 1 cm × 1 cm) was fixed in Bouin’s solution for 24 h and then stored in 75% alcohol for histology analysis. The rest of the liver tissues were snap-frozen and placed in liquid nitrogen for biochemical, gene expression, and metabolomic detection.

### 2.5. Biochemical Analysis

The physiological saline was added into the weighed liver at a ratio of volume (mL)/weight (g) = 1:9, and this was homogenized and centrifuged at 2500 rpm at 4 °C for 10 min. Then, the supernatant was collected and diluted with physiological saline to prepare a 1% tissue homogenate supernatant. The activities of active total superoxide dismutase (T-SOD, A001-3-2), catalase (CAT, A007-1-1), glutathione peroxidase (GSH-Px, A005-1-2), and total antioxidant capacity (T-AOC, A015-2-1) in the liver tissues were assayed using test kits (Nanjing Jiancheng Bioengineering Institute, Nanjing, China).

### 2.6. Quantitative Real Time-PCR (qRT–PCR) Analysis

The total RNA was extracted from the liver using the FastPure Cell/Tissue Total RNA Isolation Kit V2 (Vazyme biotech. Co., Ltd., Nanjing, China). To assess the integrity of RNA, 1 μL RNA solution was loaded into a 1% agarose gel for electrophoresis. The RNA concentration was measured by a NanoDrop One UV spectrophotometer (Thermo Fisher, Waltham, MA, USA). The HiScript III All-in-one RT SuperMix Perfect for qPCR kit (Vazyme biotech Co., Ltd., Nanjing, China) was used to obtain cDNA from reverse transcription of 1 μg RNA.

The qRT–PCR analysis was performed via the Applied Biosystems^®^ QuantStudio™ 6 Flex Real-Time PCR System (ABI, Waltham, MA, USA) using iTaq Universal SYBR Green Supermix (Bio-Rad, Hercules, CA, USA) as the fluorescent reagent. The qRT–PCR primers for superoxide dismutase 1 (*Sod1*), superoxide dismutase 2 (*Sod2*), catalase (*Cat*), glutathione peroxidase 3 (*Gpx3*), and glutathione peroxidase 4 (*Gpx4*) were designed by Primer Premier 6.0 software and are listed in Table S1. The *Efα1* was used as the reference gene. The qRT–PCR reaction mixture consisted of 10 μL of 2 × SYBR Green Supermix, 1 μL of 10 μM forward and reverse primers, 200 ng of cDNA, and ultrapure water to a final volume of 20 μL. The qRT–PCR program was run as follows: 95 °C for 5 min, then 40 cycles of 95 °C for 5 s, then 60 °C for 30 s. For each experimental group, five biological replicates, which were run in three technical duplicates per sample, were used to decrease individual differences and technique errors. The gene expression levels in the experimental groups relative to the control group were calculated using the 2^−ΔΔCt^ method [21].

### 2.7. Histological Analysis

Liver tissues were fixed in Bouin’s for 24 h and stored in 75% alcohol for histopathological examination. After dehydration with concentration gradients ethanol and transparency by xylene, samples were embedded in paraffin and sectioned (5 μm). Sections were spread on glass slides and stained in hematoxylin and eosin (H&E). The Nikon eclipse Ti2 transmission electron microscope (Nikon Corporation, Tokyo, Japan) was used to observe the histological images.

### 2.8. Metabolomic Analysis of Liver

The liver tissues in the CG, TA2, LTCG (CG challenged with 15 °C), and LTTA2 (TA2 challenged with 15 °C) were chosen for metabolomic analysis (n = 5). Metabolites were extracted from 50 mg of liver samples using a mixed solution (methanol/acetonitrile/water = 2: 2: 1) containing an internal standard (L-2-chlorophenylalanine, 1 ppm). The liquid mixtures were vortexed for 30 s, homogenized at 45 Hz for 10 min, sonicated for 10 min, and incubated on ice for 15 min. After freezing at −20 °C for one hour, the samples were centrifuged at 12,000× *g* for 15 min at 4 °C. The 60 μL supernatants were analyzed by the liquid chromatography–tandem mass spectrometry (LC–MS) system. The LC–MS system used Waters Acquity I-Class PLUS UHPLC coupled with a Waters Xevo G2-XS QT mass spectrometer. The Waters Acquity UPLC HSS T3 column was used in the LC with 2 μL injection volume. In positive and negative ion modes, mobile phase A was 0.1% formic acid aqueous solution; meanwhile, mobile phase B was 0.1% formic acid acetonitrile. In each data acquisition cycle, dual-channel data acquisition could be performed on both low collision energy and high collision energy at the same time. Low collision energy is off, high collision energy range is 10~40 V, and scanning frequency is 0.2 s for a mass spectrum. Metabolite separation was performed by UHPLC, where primary and secondary mass spectra were obtained using MassLynx V4.2 software. Electrospray ionization (ESI) was used for each sample.

The procession of raw data included peak extraction, alignment, and quantification with Progenesis QI software (Waters Corporation, Milford, MA, USA). Metabolites were identified via public databases. The orthogonal partial least squares discriminant analysis (OPLS–DA) for multivariate analysis was performed using the R language package. Cross-validation and permutation tests were conducted to verify the reliability and repeatability of the OPLS–DA model. Differentially expressed metabolites (DEMs) in the CG vs. TA2, CG vs. LTCG, TA2 vs. LTTA2, and LTCG vs. LTTA2 pairwise comparisons were filtered by |log2FC| > 1, VIP > 1, and *p* < 0.05. To explore the enriched pathways, the DEMs were annotated via the Kyoto Encyclopedia of Genes and Genomes (KEGG) public database.

### 2.9. Statistical Analysis

The plasma biochemical data were shown as mean ± standard error (SE). As for the appropriate statistical methods, the normality of distribution was assessed by the Shapiro–Wilk test, and Levene’s test, for all data, assessed the homogeneity of variances. Significant difference in plasma biochemical and gene expression results in five groups at the same time-point was evaluated by two-way analysis of variance (ANOVA) followed by Duncan’s post hoc test. *p* < 0.05 was considered to be statistically significant. IBM SPSS 21.0 software (Armonk, New York, NY, USA) was utilized for statistical analysis.

## 3. Results

### 3.1. Effect of TA on Cumulative Mortality During Feeding Trial

**Figure 2:**
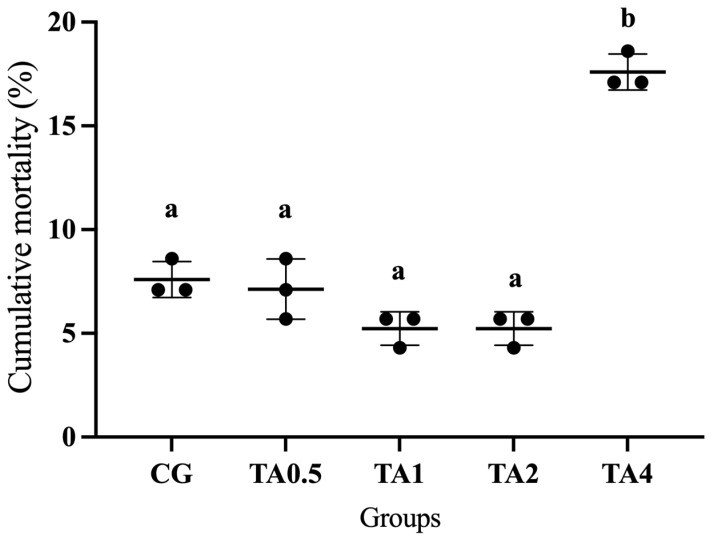
Tannic acid dosage affects cumulative mortality of the Chinese soft-shelled turtle after 60-day administration. The different letters indicates significant differences among groups (*p* < 0.05). CG indicates the control group. TA0.5 indicates the group supplemented with 0.5 g/kg tannic acid. TA1 indicates the group supplemented with 1 g/kg tannic acid. TA2 indicates the group supplemented with 2 g/kg tannic acid. TA4 indicates the group supplemented with 4 g/kg tannic acid.

To study the toxic effect of TA on the Chinese soft-shelled turtle, cumulative mortality after the 60-day administration was recorded. As shown in Figure 2, cumulative mortality in the CG and TA0.5 was 7.60% and 7.13%, respectively, which was followed by 5.23% in both TA1 and TA2. Cumulative mortality reached 17.60% in the TA4, significantly higher than the other four groups (*p* < 0.05). Generally, TA concentration from 0 g/kg to 2 g/kg had no significant difference on cumulative mortality, but 4 g/kg TA significantly increased it (*p* < 0.05).

### 3.2. Effect of TA on Hepatic Antioxidant Capacity

**Figure 3:**
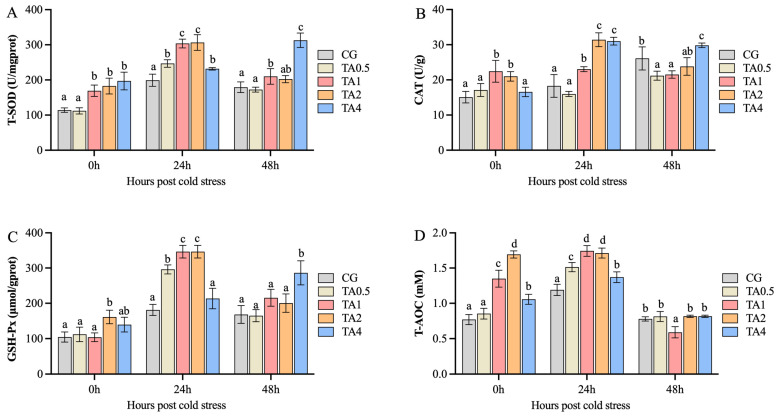
The effect of tannic acid on activities of hepatic antioxidant enzymes during temperature fluctuation, including T-SOD (**A**), CAT (**B**), GSH-Px (**C**), and T-AOC (**D**). All data are represented as mean ± SE (n = 3). Different letters indicate significant differences among groups at the same time points (*p* < 0.05). T-SOD, total superoxide dismutase (U/mgprot); CAT, catalase (U/g); GSH-Px, glutathione peroxidase, (µmol/mgprot); T-AOC, total antioxidant capacity (mM). CG indicates the control group. TA0.5, TA1, TA2, and TA4 respectively indicate the groups supplemented with 0.5 g/kg, 1 g/kg, 2 g/kg, and 4 g/kg tannic acid. 0 h indicates the endpoint of the 60-day administration. 24 h indicate a 24 h post 15 °C cold stress. 48 h indicates 24 h after returning to 28 °C.

To evaluate the influence of TA on antioxidant capacity, the activities or contents of T-SOD, CAT, GSH-Px, and T-AOC in the liver were measured (Figure 3). Hepatic T-SOD activity (U/mgprot) and T-AOC (mM) in the TA1, TA2, and TA4 groups were significantly higher (*p* < 0.05) than those in the CG and TA0.5 at 0 hps and 24 hps. Their activity in the TA4 was significantly higher (*p* < 0.05) than in the other groups at 48 hps. CAT activity (U/g) in TA1 and TA2 was improved at 0 hps and 24 hps, while it significantly increased (*p* < 0.05) in the TA4 at 24 hps and 48 hps compared with the CG. GSH-Px activity (μmol/gprot) was significantly higher in the TA2 than in the other four groups at 0 hps (*p* < 0.05); meanwhile, it significantly increased in the TA0.5, TA1, and TA2 at 24 hps, as well as in the TA4 at 48 hps (*p* < 0.05).

**Figure 4:**
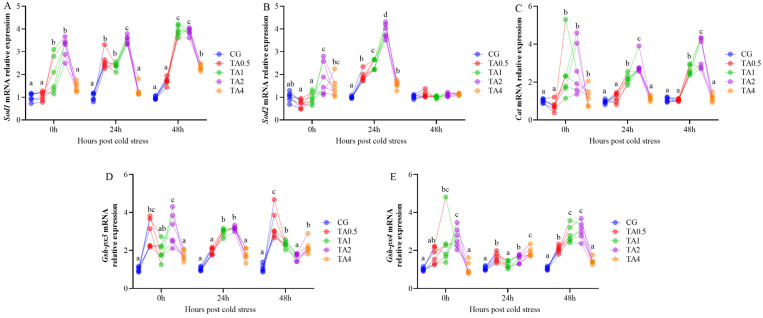
The effect of tannic acid on mRNA expression of hepatic antioxidant enzymes during temperature fluctuation, including *Sod1* (**A**), *Sod2* (**B**), *Cat* (**C**), *Gsh-px3* (**D**), and *Gsh-px4* (**E**). The different letters indicate significant differences among groups at the same time points (*p* < 0.05). *Sod1*, total superoxide dismutase 1; *Sod2*, total superoxide dismutase 2; *Cat*, catalase; *Gsh-px3*, glutathione peroxidase 3; *Gsh-px4*, glutathione peroxidase 4. CG indicates the control group. TA0.5, TA1, TA2, and TA4, respectively, indicate the groups supplemented with 0.5 g/kg, 1 g/kg, 2 g/kg, and 4 g/kg tannic acid. 0 h indicates the endpoint of the 60-day administration. 24 h indicates 24 h post 15 °C cold stress. 48 h indicates 24 h after returning to 28 °C.

The *Sod1*, *Sod2*, *Cat*, *Gsh-px3*, and *Gsh-px4* mRNA expression in the TA2 was significantly more abundant than in the other groups at 0 hps (*p* < 0.05) (Figure 4). At 24 hps, the *Sod1*, *Sod2*, *Gsh-px3*, and *Gsh-px4* mRNA levels in the TA0.5, TA1, and TA2 were up-regulated compared with the CG. The *Sod1*, *Cat*, and *Gsh-px4* mRNA levels in the TA1, TA2, and TA4 were significantly higher than that in the CG and TA0.5 at 48 hps (*p* < 0.05).

### 3.3. Effect of TA on Hepatic Damage

**Figure 5:**
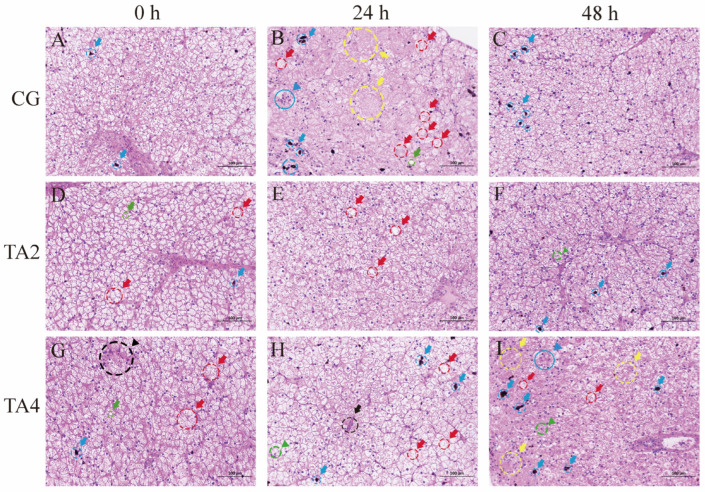
The hepatic histology of the Chinese soft-shelled turtle in three TA dosage groups during temperature fluctuation. The hepatic histology in CG at 0 h (**A**), 24 h (**B**), and 48 h (**C**). The hepatic histology in TA2 at 0 h (**D**), 24 h (**E**), and 48 h (**F**). The hepatic histology in TA4 at 0 h (**G**), 24 h (**H**), and 48 h (**I**). CG indicates the control group. TA2 and TA4, respectively, indicate the groups supplemented with 2 g/kg and 4 g/kg TA. 0 h indicates the endpoint of the 60-day administration. 24 h indicates 24 h post-15 °C cold stress. 48 h indicates 24 h after returning to 28 °C. Scale bar = 100 μm. The black arrow indicates that hepatic sinuses were enlarged and congested with erythrocytes. The yellow, red, green, and blue arrows, respectively, represent the cell necrosis, cell edema, steatosis, and hemosiderin deposition. The black, green, and blue triangles, respectively, point out the acidophilic bodies, nuclear migration, nuclear deformation, and nuclear swelling.

As shown in Figure 5, H and E staining was applied to detect the influence of TA on the histopathological changes in the liver. After the 60-day feeding trial, the hepatocytes in the CG and TA2 were in a healthy state, exhibiting normal round nuclei, a clear and complete hepatic cord, and narrow hepatic sinuses. A few hemosiderin depositions (Figure 5A,D, blue arrows), edema (Figure 5A,D, red arrows), and steatosis (Figure 5D, green arrow) were found in the CG and TA2. In contrast, the boundary of a few hepatocytes became obscure in the TA4; moreover, nuclear migration (Figure 5G, black triangle) was observed in some hepatocytes in the TA4. At 24 hps, the hepatocyte boundary became obscure in the CG (Figure 5B); however, it kept clear in the TA2 and TA4. Furthermore, edema (Figure 5B), hemosiderin depositions (Figure 5B), steatosis (Figure 5D), and nuclear swelling (Figure 5B, blue triangle) were found in the CG. Meanwhile, the hepatocytes in the TA4 representing the hepatic sinus were enlarged and congested with erythrocytes (Figure 5H, black arrow). Different from these two groups, a few edemas (Figure 5E), and no hemosiderin deposition (Figure 5B,H) or steatosis (Figure 5B) were detected in the TA2. At 48 hps, the hepatocyte boundary remained distinct in the TA2 and TA4, but had severely disappeared in the CG (Figure 5C, yellow arrow). Furthermore, large-scale hepatocyte necrosis, edema (Figure 5C,I), steatosis (Figure 5C,I), increased quantities of hemosiderin deposition (Figure 5C,I), and nuclear deformation (Figure 5I, green triangle) were exhibited in the CG and TA4. However, the injuries in the TA2 at 48 hps were relatively slight, mainly including quantities of hemosiderin deposition (Figure 5F) and nuclear deformation (Figure 5F). In general, 15 °C cold stress caused hepatocyte injuries in the CG and TA4, which were alleviated in the TA2. Furthermore, these injuries in the three groups were aggravated at 24 h after returning to 28 °C.

### 3.4. Metabolite Profiles Affected by TA and Low Temperature

Comparative metabolomic analysis was applied to reveal the metabolic profiles in the liver responding to dietary TA and low temperature. Four comparisons, including CG vs. TA2, CG vs. LTCG, TA vs. LTTA2, and LTCG vs. LTTA2 were analyzed to select the metabolites affected by TA and temperature. Specifically, CG vs. TA2 and LTCG vs. LTTA2 comparisons, respectively, evaluated the variations induced by dietary TA at 28 °C and 15 °C. In addition, CG vs. LTCG and TA vs. LTTA2 comparisons separately assessed the effect of low temperature on the metabolic profiles of CG and 0.2% TA.

**Figure 6:**
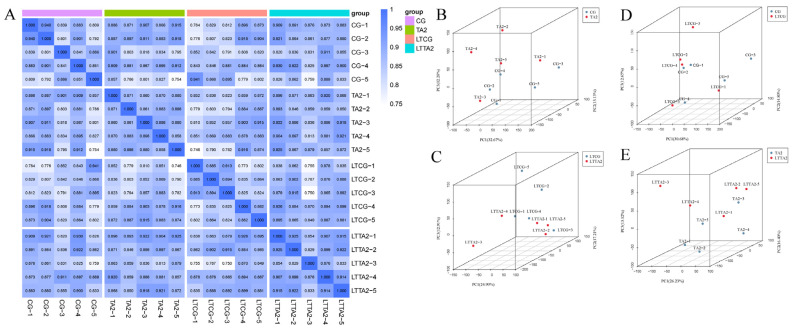
Evaluation of metabolomic quality. (**A**) Correlation heatmap of all metabolites in four groups. (**B**–**E**) Principal component analysis (PCA) exhibits the difference in metabolic patterns in four pairwise comparisons, including CG vs. TA2 (**B**), LTCG vs. LTTA2 (**C**), CG vs. LTCG (**D**), and TA2 vs. LTTA2 (**E**). CG indicates the control group. TA2 indicates the 2 g/kg tannic acid group. LTCG indicates low temperature-stressed CG. LTTA2 indicates low temperature-stressed TA2.

The correlation matrix with Pearson’s correlation coefficients can evaluate correlations of the biological repeats in the same group (Figure 6A). Most of the PCC values were more than 0.8, implying the good repeatability of the five replicates in the same group. Principal component analysis (PCA) can represent the relationship between different groups (Figure 6B–E). Our PCA results clearly distinguished each of the four pairwise comparisons. Specifically, the first principal component (PC1) and second principal component (PC2) separately explained 32.67% and 13.13% of the variance in the CG vs. TA2 (Figure 6B). Meanwhile, PC1 and PC2 accounted for 24.90% and 17.21% of the total variance (Figure 6C) in the LTCG vs. LTTA2. These indicated that the samples in the TA group could be discriminated when compared to those in the control group. In addition, the PC1 values were 30.68% and 26.23%, respectively, in the CG vs. LTCG (Figure 6D) and TA vs. LTTA2 (Figure 6E), which indicated that the samples could be differentiated by the low temperature.

**Figure 7:**
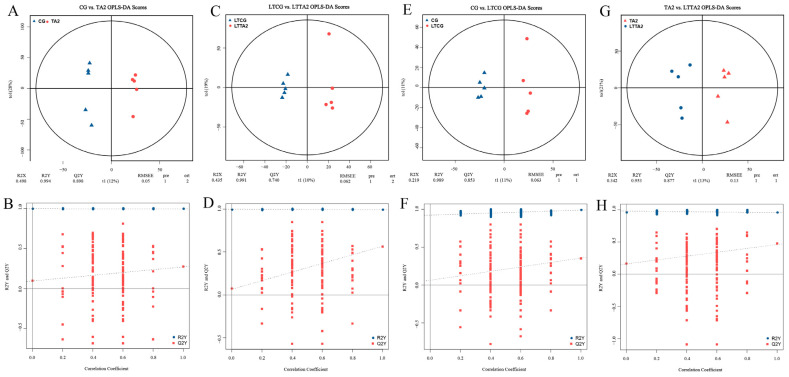
Multivariate statistical analysis of metabolome in different groups. (**A**,**C**,**E**,**G**) Orthogonal projection of latent structures–discriminant analysis (OPLS–DA) score plots in four pairwise comparisons, including CG vs. TA2 (**A**), LTCG vs. LTTA2 (**C**), CG vs. LTCG (**E**), and TA2 vs. LTTA2 (**G**). Permutation tests of the OPLS–DA models for the CG vs. TA2 (**B**), LTCG vs. LTTA2 (**D**), CG vs. LTCG (**F**), and TA2 vs. LTTA2 (**H**). “R2Y” and “Q2Y”, respectively, indicate the explanatory rate and the predictive ability of the OPLS–DA model. CG indicates the control group. TA2 indicates the 2 g/kg tannic acid group. LTCG indicates low temperature-stressed CG. LTTA2 indicates low temperature-stressed TA2. The dotted lines indicated the regression lines fitted by R2Y (blue) and Q2Y (red). The solid lines indicated the zero line.

To reveal the different metabolites between the pairwise groups, multivariate statistical analysis was accomplished with the OPLS–DA model. The OPLS–DA model was verified with cross-validation and permutation tests (Figure 7). The R2Y > 0.96 and Q2Y > 0.50 of cross-validation showed the goodness of fit and high predictability of the OPLS–DA model (Figure 7A,C,E,G). The R2Y = 0.994 and Q2Y = 0.898 in the CG vs. TA2 (Figure 7A), R2Y = 0.991 and Q2Y = 0.740 in the LTCG vs. LTTA2 (Figure 7C), R2Y = 0.989 and Q2Y = 0.853 in the CG vs. LTCG (Figure 7E) as well as R2Y = 0.953 and Q2Y = 0.877 in the TA2 vs. LTTA2 (Figure 7G), indicated the stability of the OPLS–DA models in the four comparisons. Permutation tests were used to examine the accuracy of the OPLS–DA models (Figure 7B,D,F,H). The Y-intercept of Q2 values in the permutation tests were all lower than 0.2, showing the low risk of overfitting and good reliability of the OPLS–DA models. Therefore, our OPLS–DA models were stable and reliable in detecting metabolic differentiation in different groups, and found an apparent distinction in the four pairwise comparisons. Based on the OPLS–DA models, the VIP > 1 and *p* < 0.05 were set to screen the DEMs in pairwise comparisons.

**Figure 8:**
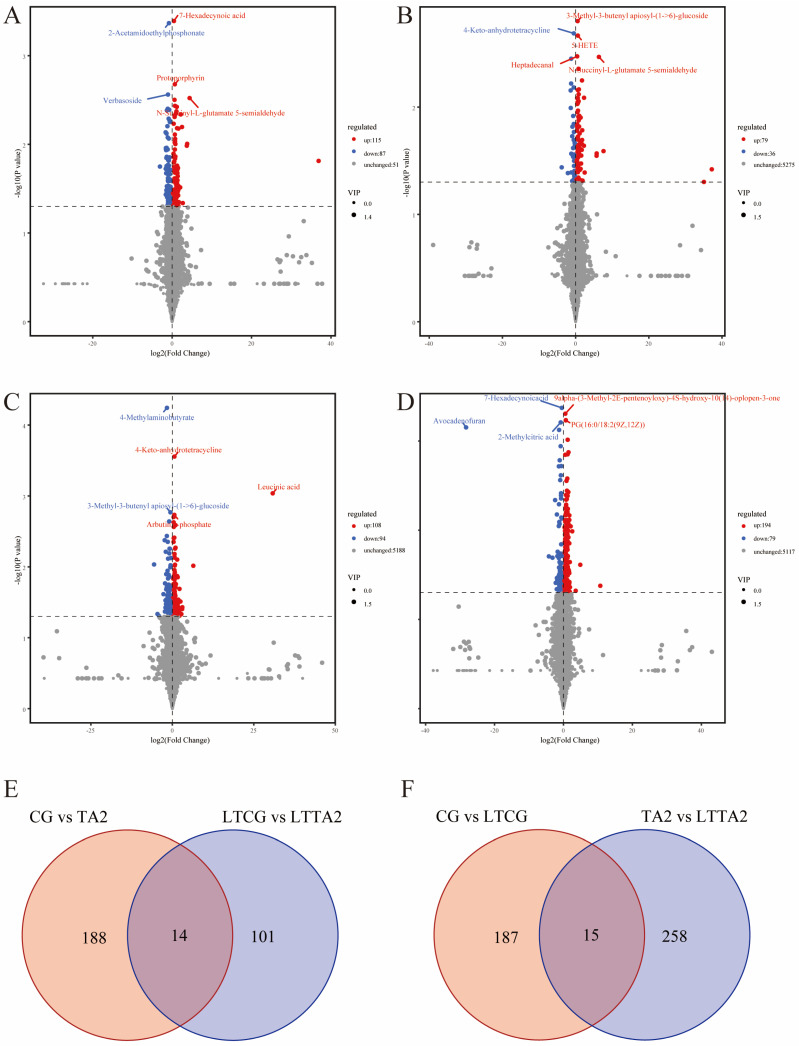
The number of differentially expressed metabolites (DEMs) in four comparisons, including CG vs. TA2 (**A**), LTCG vs. LTTA2 (**B**), CG vs. LTCG (**C**), and TA2 vs. LTTA2 (**D**). CG indicates the control group. TA2 indicates the 2 g/kg tannic acid group. LTCG indicates low temperature-stressed CG. LTTA2 indicates low temperature-stressed TA2. The Venn diagram found a common DEM number in different comparisons, including CG vs. TA2 and LTCG vs. LTTA2 (**E**), as well as CG vs. LTCG and TA2 vs. LTTA2 (**F**). CG indicates the control group. TA2 indicates the 2 g/kg tannic acid group. LTCG indicates low temperature-stressed CG. LTTA2 indicates low temperature-stressed TA2.

The volcano maps were plotted to intuitively show the quantities of DEMs in the four pairwise comparisons (Figure 8). A total of 202 DEMs (115 up-regulated and 87 down-regulated) in the CG vs. TA2 (Figure 8A), 115 DEMs (79 up-regulated and 36 down-regulated) in the LTCG vs. LTTA2 (Figure 8B), 202 DEMs (108 up-regulated and 94 down-regulated) in the CG vs. LTCG (Figure 8C), and 273 DEMs (194 up-regulated and 79 down-regulated) in the TA vs. LTTA2 (Figure 8D), respectively, were detected by the comparative metabolome. Furthermore, the Venn plot exhibited a total of 14 DEMs in both CG vs. TA2 and LTCG vs. LTTA2 comparisons (Figure 8E); meanwhile, there were 15 common DEMs in both CG vs. LTCG and TA2 vs. LTTA2 comparisons (Figure 8F).

**Figure 9:**
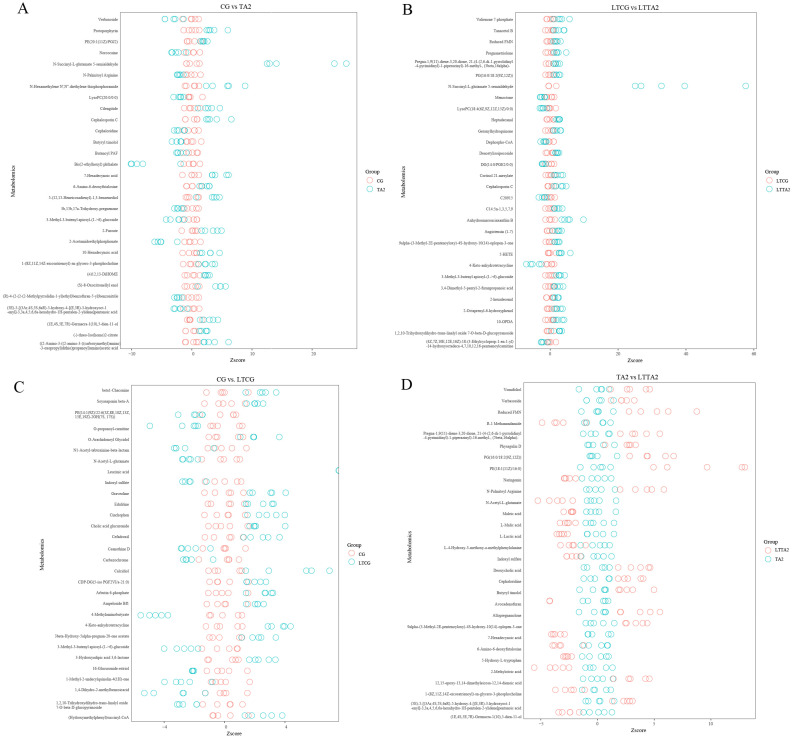
Z-score plots exhibiting the top 30 differentially expressed metabolites (DEMs) in four comparisons, including CG vs. TA2 (**A**), LTCG vs. LTTA2 (**B**), CG vs. LTCG (**C**), and TA2 vs. LTTA2 (**D**). CG indicates the control group. TA2 indicates the 2 g/kg tannic acid group. LTCG indicates low temperature-stressed CG. LTTA2 indicates low temperature-stressed TA2.

The Z-score plots showed the top 30 DEMs ranked by *p* values in the pairwise comparison (Figure 9). In the CG vs. TA2, N-Succinyl-L-glutamate 5-semialdehyde, N-Hexamethylene N′, N″-diethylene thio-phosphoramide, ((2-amino-3-((2-amino-3-((carboxymethyl)amino)-3-oxopropyl) dithio) propanoyl) amino) acetic acid, cephalosporin C, and protoporphyrin were the top five most increased DEMs, while bis(2-ethylhexyl) phthalate, 2-acetamidoethylphosphonat, verbasosid, norcocain, and 3-Methyl-3-butenyl apiosyl-glucoside obviously declined (Figure 9A and Table S2). Furthermore, various kinds of lipids and lipid-like molecules were increased in the CG vs. TA2, such as 25-hydroxy-24-oxocholecalciferol, 17,18-EpETE, PS(14:1(9Z)/18:4(6Z,9Z,12Z,15Z)), hydratopyrrhox-anthinol, L-menthyl (R,S)-3-hydroxybutyrate, trans-hexa-dec-2-enoic acid, myrtenyl acetate, and sorbitan laurate.

In the LTCG vs. LTTA2, N-succinyl-L-glutamate 5-semialdehyde, anhydro-amaroucia-xanthin B, valienone 7-phosphate, 5-HETE, and pregnanetriolone were the top five most enhanced DEMs while 4-keto-anhydrotetracyclin, dephospho-CoA, menoctone, lysoPC(18:4(6Z,9Z,12Z,15Z)/0:0), and (4Z,7Z,10E,12E,16Z)-18-(3-ethylcycloprop-1-en-1-yl)-14-hydroxyoctadeca-4,7,10,12,16-pentaenoylcarnitine had obviously decreased (Figure 9B and Table S3). In the CG vs. LTCG, calcidiol, 4-keto-anhydrotetracycline, (hydroxy-methylphenyl) succinyl-CoA, cinchophen, and cefadroxil were the top five most increased DEMs, while 4-methylaminobutyrate, 1,4-dihydro-2-methylbenzoicacid, O-propanoyl-carnitine, 3-methyl-3-butenyl apiosyl-glucoside, and 1-methyl-2-undecylquinolin-4(1H)-one had obviously declined (Figure 9C and Table S4). In the TA vs. LTTA2, PE (18:1(11Z)/16:0), FMN, pg(16:0/18:2(9Z,12Z)), N-palmitoyl arginine, and allopregnanolone were the top five most increased DEMs, while 2-methylcitric acid, N-acetyl-L-glutamate, R-1 methanandamide, avocadenofuran, and 7-hexadecynoic acid had most obviously declined (Figure 9D and Table S5).

There were 14 common DEMs both in CG vs. TA2 and LTCG vs. LTTA2 (Table 2), such as deoxycholic acid, phenyl-propanoic acid, hypogeic acid, 7-hexadecynoic acid, 3-methyl-3-butenyl apiosyl-(1->6)-glucoside, and N1-acetyl-tabtoxinine-beta-lactam. These DMEs might be potential metabolites, resulting in the difference between CG and TA2 at 28 °C and 15 °C.

**Figure 10:**
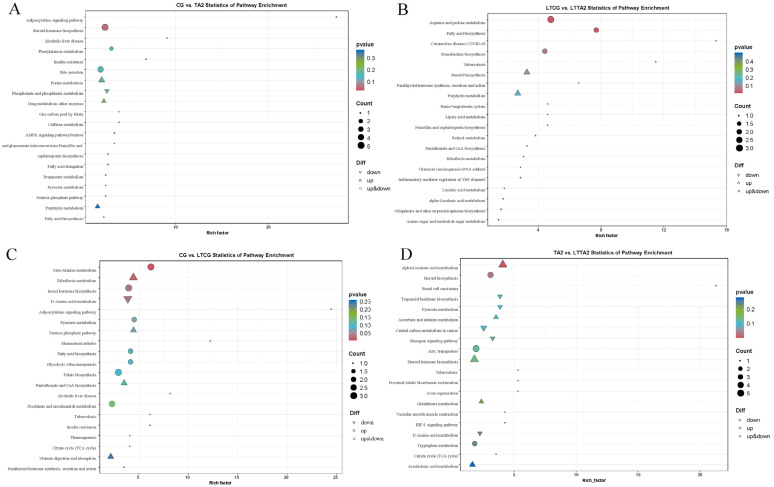
KEGG analysis exhibiting the top 20 enrichment pathways of differentially expressed metabolites (DEMs) in four comparisons, including CG vs. TA2 (**A**), LTCG vs. LTTA2 (**B**), CG vs. LTCG (**C**), and TA2 vs. LTTA2 (**D**). CG indicates the control group. TA2 indicates the 2 g/kg tannic acid group. LTCG indicates low temperature-stressed CG. LTTA2 indicates low temperature-stressed TA2.

The KEGG analysis of the DEMs obtained 42, 25, 47, and 54 pathways, respectively, in the CG vs. TA2, CG vs. LTCG, LTCG vs. LTTA2, and TA2 vs. LTTA2 pairwise comparisons. The “adipocytokine signaling pathway”, “insulin resistance”, “one carbon pool by folate”, “caffeine metabolism”, “AMPK signaling pathway”, “pentose and glucuronate interconversions”, and “phenylalanine metabolism” were the top eight pathways in the CG vs. TA2 (Figure 10A and Table S6), while “fatty acid biosynthesis”, “parathyroid hormone synthesis, secretion and action”, “arginine and proline metabolism”, “monobactam biosynthesis”, “renin–angiotensin system”, and “linoleic acid metabolism” were most abundant in the LTCG vs. LTTA2 (Figure 10B and Table S7). Furthermore, the “adipocytokine signaling pathway”, “beta-alanine metabolism”, “insulin resistance”, “pyruvate metabolism”, and “riboflavin metabolism” were remarkably enriched in the CG vs. LTCG (Figure 10C and Table S8), while “proximal tubule bicarbonate reclamation”, “alpha-linolenic acid metabolism”, “vascular smooth muscle contraction”, “terpenoid backbone biosynthesis”, and “pyruvate metabolism” were most abundant in the TA2 vs. LTTA2 (Figure 10D and Table S9).

## 4. Discussion

Until now, much research has comprehensively studied the effect of TAs on the intestinal health of many aquatic species, such as *Lateolabrax maculatus* [22], *Micropterus salmoides* [11], and *Penaeus vannamei* [23]. As the liver is the predominant organ coping with external stimuli, it is important to explore the effect of TA on hepatic function. However, a few studies have reported that TA affects liver health, including the histomorphology of the hepatopancreatic condition in the Pacific White Shrimp [24], lipid metabolism in the Japanese seabass [25], and hepatic antioxidant capacities in Chinese Seabass [26]. The role of TA in regulating hepatic health under physiological stressors is still rare in the Chinese soft-shelled turtle. Therefore, revealing the effect of TA on hepatic histology, antioxidant capacities, and metabolic mechanisms under low temperatures can extend the knowledge of TA’s function in resisting cold stress for reptiles.

### 4.1. Cumulative Mortality and Histological Injury

TA has harmful and beneficial effects depending on its nature, concentration, animal species, animal health, and feed composition [27]. More than 5% levels of TA can reduce feed intake, and above 9% TA causes mortality in the animal [28]. Previous research has found that calves fed low dosage TA were kept in a healthy state; however, more than 4400 mg/kg TA induced methaemoglobinemia and even death in the calves [29]. Supplementation of more than 10,000 mg/kg TA reduced feed intake and egg production [27]. Cumulative mortality is an important index to evaluate the toxicity of dietary TA on animals. TA exhibits detrimental effects on animals, mainly via negatively affecting the feeding, growth, and development of animals or insects [30]. These current results showed that 4 g/kg TA significantly elevated cumulative mortality during the 60-day feeding trial, which might be attributed to the toxic property of TA.

Histopathology is a typical technique used to evaluate the health status of organs or tissues, and has been widely utilized in studying the effect of external stimuli on tissue damage. For example, histopathological detection has observed that hepatic cells exhibited various injuries in the Chinese soft-shelled turtle, with white abdominal disease, consisting of a thickened arterial wall, inflammatory cell infiltration, and blurred cell boundaries [31]. In the current research, histology analysis found that the liver tissue was slightly injured in the TA4 after 60 days of feeding trial, with blurred cell boundaries. Furthermore, exposure to 15 °C for 24 h induced multiple hepatic damages in CG, including the obscure hepatocyte boundary, large-scale hepatocyte necrosis, steatosis, increased hemosiderin deposition, and edema, which were alleviated in the TA2 but appeared more severely in the TA4. When the temperature returned to 28 °C, the hepatic injuries were mitigated in the CG vs TA2, but worsened in the TA4. Similarly, analysis of hepatopancreas histology in the shrimp found that TA can promote hepatopancreas morphology by improving the tubular structure and lumen star-shaped morphology [24]. Low dosages of TA from 0.2 g/kg to 1 g/kg do not affect hepatic histomorphology in Japanese seabass [25]. In addition, similar research in Chinese seabass has shown that dietary TA less than 1 g/kg does not influence liver morphology. In contrast, a dosage over 1 g/kg can damage the liver, as represented by obvious vacuolar degeneration, inflammatory cell infiltration, and increased eosinophilic infiltrate and necrosis [26]. In general, our results suggest that TA could regulate hepatic histomorphology dose-dependently. The negative effect of 4 g/kg TA on hepatic histomorphology was in accordance with the higher cumulative mortality. Meanwhile, the 2 g/kg TA effectively mitigated the hepatic injuries caused by cold stress in the Chinese soft-shelled turtle. This is the first report demonstrating that TA can protect the hepatic morphology from cold stress-induced damage.

### 4.2. Antioxidant Response of Liver

Cold stress can induce the excessive production of reactive oxygen species, which are detrimental to various crucial biological molecules, such as DNA, proteins, and lipids, i.e., oxidative stress [32]. To clear the accumulated ROS, antioxidant defense systems are triggered, such as via the activation of antioxidant enzymes. SOD, CAT, and GSH-Px, as the primary antioxidant enzymes, are involved in removing the ROS and protecting the organism from oxidative stress damage [33]. In the present study, SOD is commonly considered as an indicator to evaluate the ability to respond to oxidative stress, which can catalyze dismutation O_2_^.-^ into O_2_ and H_2_O_2_ in the mitochondrial intermembranous space and mitochondrial matrix. H_2_O_2_ was subsequently was cleaved into H_2_O and O_2_ by CAT [34]. Meanwhile, GSH-Px catalyzes the transformation between glutathione (GSH) and glutathione disulfide (GSSG), during which the H_2_O_2_ is transformed to H_2_O and lipid hydroperoxides are converted to alcohol [35].

Antioxidant enzymes play a protective role in the liver by scavenging free radicals and maintaining the oxidative/antioxidative balance. When this balance is disrupted, it leads to oxidative stress [36]. Oxidative stress is crucial in liver disease progression, including liver fibrosis, cirrhosis, and hepatocellular carcinoma [37]. TA can remove oxygen-free radicals in animals, thus improving antioxidant enzyme activity in order to reduce oxidative stress [38]. In this study, we found that the 1 g/kg and 2 g/kg TA significantly increased the activities of SOD, CAT, and T-AOC as well as improving the mRNA expression of *Sod1*, *Cat*, and *Gsh-px4* after 60-day administration and 15 °C cold stress, which returned to its initial levels after 24 h at 28 °C. Although 4 g/kg TA also improved the SOD activity and T-AOC, the increment of the antioxidant enzymes was smaller than that at the 1 g/kg and 2 g/kg dosages. The present results were consistent with the previous report on the Chinese Seabass, which showed that 0–1 g/kg TA enhanced the activities of SOD, CAT, and T-AOC [25]. Similarly, SOD, CAT, and T-AOC activities, as well as *sod* and *gpx* mRNA expression, increased by 1.50 and 3.75 g/kg TA in the largemouth bass [11]. SOD activity, as well as the mRNA levels of the *sod*, *cat*, and *gpx,* rose by 0.15% TA in the hepatopancreas of Pacific white shrimp [23]. In brief, the current results indicate that administration of 2 g/kg TA improved the hepatic antioxidant capacity of the Chinese soft-shelled turtle under cold stress, which can further protect the liver from ROS-induced injuries.

### 4.3. Regulation of TA in Metabolic Profiles

In this research, there were 202 DEMs in the CG vs. TA2. Of these DEMs, the contents of protoporphyrin, verbasoside, and N-Succinyl-L-glutamate 5-semialdehyde classification oxidoreductase were obviously changed. Protoporphyrin and verbasoside content were enhanced after 2 g/kg TA supplementation in this study. Protoporphyrin is the immediate precursor of the heme molecule, which is regularly found in small amounts in erythrocytes and excreted into the bile [39]. It has been found to be beneficial for regulating oxidative stress levels [40]. Verbasoside is a polyphenol with one catechol unit showing antioxidant activity [41]. In LPS-induced acute lung injury, verbascoside can activate NF-κB pathways to mitigate lipopolysaccharide-induced inflammation [42]. N-Succinyl-L-glutamate 5-semialdehyde classification oxidoreductase can convert from ATP and N-acetyl-L-glutamate. Previous research has found that N-Succinyl-L-glutamate 5-semialdehyde is affected by agents involved in traditional Chinese medicine [43].

The liver plays essential roles in fat metabolism, and can synthesize phospholipids and cholesterol, which are essential for hepatic production of bile salts, steroid hormones, and components of plasma membranes [44]. Herein, various kinds of DEM categorized into lipids and lipid-like molecules were improved in the TA2 compared with CG, such as 25-hydroxy-24-oxocholecalciferol, 17,18-EpETE, PS(14:1(9Z)/18:4(6Z,9Z,12Z,15Z)), hydratopyrrhoxanthinol, L-menthyl (R,S)-3-hydroxybutyrate, trans-hexa-dec-2-enoic acid, myrtenyl acetate, and sorbitan laurate. These results hinted that 2g/kg TA might promote liver function in lipid metabolism, which can help animals to adjust to environmental stressors, such as low temperature [45]. DEMs in the CG vs. TA2 were enriched in the “adipocytokine signaling pathway,” “AMPK signaling pathway”, “bile secretion”, and “fatty acid biosynthesis”. The adipocytokine signaling pathway contains a variety of hormones and cytokines synthesized and secreted by fat cells, which are involved in energy metabolism, inflammatory response, and immunity [46]. AMP-activated protein kinase (AMPK) is an important energy sensor within cells, capable of monitoring the ratio of ATP to AMP in order to maintain energy homeostasis in eukaryotic cells [47]. In our study, the adipokine signaling pathway and AMPK signaling pathway were activated in the CG vs. TA2 comparison. A similar report in Brandt’s Voles has found that TA treatment can increase the adiponectin level and glucose uptake by activating the AMPK/GLUT1 pathway [48]. In general, current research indicates that 2 g/kg TA might promote liver function in the secretion of bile, inflammatory response, and energy metabolism by regulating the above metabolites or signaling pathways. Furthermore, considering the influence of 2 g/kg TA on lipid-related metabolites, we hypothesized that 2 g/kg TA might be helpful for turtles in dealing with cold stress by regulating cellular membrane fluidity.

To verify the metabolic mechanism of TA in responding to cold stress, it is necessary to perform a comparative metabolome regarding LTCG vs. LTTA2. The DEMs and pathways in the LTCG vs. LTTA2 comparison explained the potential mechanism of TA in coping with cold stress. Herein, 115 DEMs were identified in the LTCG vs. LTTA2 comparison. Of these DEMs, leucinic acid has been found to be regulated by *Anoectochilus roxburghii* polysaccharide in mice [49]. Similar research in laying hens has shown that leucinic acid is involved in the function of berberine in alleviating fatty liver hemorrhagic syndrome [50]. Likewise, the leucinic acid was affected by the TA in this research, which indicated that leucinic acid might be a sign of the feed additive reshaped metabolism and function. Cellular membrane properties, such as integrity and temperature-compatible fluidity, are important for maintaining cellular functions and enhancing survival under cold stress. Glycerophospholipids are the main constitutive lipids on the cellular membrane, whose unsaturation level and acyl chain length can affect the membrane fluidity [51]. It is noteworthy that three types of glycerophospholipids, containing PC(18:2(9Z,12Z)/20:4(8Z,11Z,14Z,17Z)), 1-Palmitoyl-2-linoleoyl-sn-glycero-3-phosphate, and PG(16:0/18:2(9Z,12Z)), were enhanced in the LTTA2 liver; whereas hepatic linoleic acid and hypogeic acid, classified as unsaturated fatty acids, were declined in LTTA2. The current results indicate that 2g/kg TA was likely to maintain the fluidity of the hepatic membrane by regulating the pertinent glycerophospholipid contents under cold stress. An appropriate fluidity of the cell membrane can keep the liver function normal and stable under cold stress.

Further analysis of the DEMs in the CG vs. TA2 and LTCG vs. LTTA2 comparisons revealed 14 common DEMs in both comparisons. These DEMs include 3-methyl-3-butenyl apiosyl-(1->6)-glucoside, N-Succinyl-L-glutamate 5-semialdehyde, N1-Acetyl-tabtoxinine-beta-lactam, deoxycholic acid, phenyl-propanoic acid, hypogeic acid, and 7-hexadecynoic acid. The 14 DEMs in the current research might be important metabolites associated with the role of TA in regulating liver function at 28 °C and 15 °C. The DEMs in the LTCG vs. LTTA2 were mainly clustered in the “fatty acid biosynthesis”, “arginine and proline metabolism”, “steroid biosynthesis”, and “linoleic acid metabolism” pathways. Arginine and proline metabolism play important roles in liver health and protection. Arginine availability, synthesis, and catabolism are associated with immune responses, and their fine-tuning can affect immune outcomes [52]. It has been reported that L-arginine has a protective role in rat liver after transplantation [53]. Proline, a non-essential amino acid, is involved in the maintenance of cellular redox homeostasis. Proline has been shown to protect the liver from D-galactosamine (GalN)-induced liver injury by activating the ROS-eliminating pathway [54]. Fatty acid synthesis takes place in the cytosol and is essential for the reversing of fatty acid β-oxidation. Fatty acid synthesis involves the de novo assembly of acetate into saturated fatty acids, as well as the desaturation and elongation of dietary essential fatty acids—linoleic acid (C18:2n-6) and α-linolenic acid (C18:3n-3)—to highly unsaturated 20- and 22-carbon fatty acids, which are essential to reproduction, cell differentiation, inflammation, and cognition [55]. Linoleic acid metabolism can protect hepatocytes against lipo-toxicity from linoleic acid and α-linolenic acid in rats [56]. The steroid biosynthesis pathway primarily produces steroid hormone precursors, cholesterol, and vitamin intermediaries [57]. As essential endogenic compounds, steroids regulate almost all major physiological functions, including energy metabolism and reproduction. Previous research on dietary sweet cherry anthocyanins found that the additive attenuates diet-induced hepatic steatosis in mice by improving hepatic lipid metabolism, including steroid biosynthesis and fatty acid metabolism [58]. The current data indicates that the 2 g/kg TA might alleviate cold stress-induced liver damage by activating amino acid- and lipid-related pathways for the Chinese soft-shelled turtle.

The present research showed that TA could protect Chinese soft-shelled turtle from cold stress. In breeding, liver health was closely related to multiple factors, such as feed components and the breeding environment. Imbalanced composition of feed can affect the normal metabolism of the liver. For example, high-protein and high-fat feeds increased the digestive and absorption burden on the liver, which led to issues such as fatty liver disease [59]. Water parameters can also influence liver health. For example, nitrite affects the antioxidant system of the liver and leads to lipid peroxidation [60]. Therefore, these factors might influence the effect of TA on liver health, which needs attention during TA application.

## 5. Conclusions

This research demonstrated that not 0.5–2 g/kg, but 4 g/kg dietary TA had a toxic effect on the survival of Chinese soft-shelled turtles. During temperature fluctuation, 2 g/kg TA could effectively improve the antioxidative capacities via increasing the T-SOD, CAT, GSH-Px, and T-AOC activities, and alleviate histological injuries in the liver. Comparative metabolome analysis suggested that 2 g/kg TA might regulate energy-related signaling pathways to deal with cold stress, such as adipocytokine signaling pathway, steroid biosynthesis, and α-linolenic acid metabolism. Therefore, it is promising that the Chinese soft-shelled turtle can overcome a sudden temperature drop outdoors by supplementation with 2 g/kg TA in advance. However, a decrease in temperature in actual farming is always accompanied by alteration of water parameters, such as dissolved oxygen and ammonia concentration. This is likely to weaken the protective effect of TA for the Chinese soft-shelled turtle under cold stress.

## Figures and Tables

**Table 1:** The formulation and proximate composition of experimental diets (% of dry matter).

Ingredients	CG	TA0.5	TA1	TA2	TA4
White Fishmeal 1	40.00	40.00	40.00	40.00	40.00
White Fishmeal 2	15.00	15.00	15.00	15.00	15.00
Chicken powder	5.00	5.00	5.00	5.00	5.00
Soybean meal	9.00	9.00	9.00	9.00	9.00
patent flour	20.50	20.43	20.37	20.23	19.97
Cassava raw starch	5.00	5.00	5.00	5.00	5.00
Soybean oil	2.00	2.00	2.00	2.00	2.00
Choline chloride	1.50	1.50	1.50	1.50	1.50
NaCl	0.20	0.20	0.20	0.20	0.20
Turtle’s premix	0.20	0.20	0.20	0.20	0.20
L-lysine hydrochloride	1.00	1.00	1.00	1.00	1.00
DL-Methionine	0.40	0.40	0.40	0.40	0.40
Protein	0.20	0.20	0.20	0.20	0.20
Tannic acid	0.00	0.07	0.13	0.27	0.53
Composition	Content				
Crude protein	46.24	45.38	47.78	47.97	47.62
Crude lipid	7.65	7.26	7.61	7.51	7.21
Crude fiber	0.84	0.83	0.86	0.81	0.88
Ash	12.75	12.20	12.68	12.51	12.69
Calcium	3.35	3.15	3.36	3.18	3.25
Phosphorus	2.27	2.25	2.19	2.17	2.28

Turtles’ premix (/kg): VA, 150,000 IU; VD, 3,110,000 IU; VE, 4000 mg; VK_3_, 400 mg; VB_1_, 800 mg; VB_2_, 1450 mg; VB_6_, 2500 mg; VB_12_, 3.0 mg; VC, 16,000 mg; *D*-Calcium Pantothenate, 1250 mg; nicotinamide, 8000 mg; folic acid, 250 mg; D-biotin, 10 mg; inositol, 6000 mg; magnesium, 6000 mg; zinc, 4300 mg; manganese, 650 mg; copper, 410 mg; iron, 5900 mg; cobalt, 100 mg; iodine, 75 mg; selenium, 25 mg.

**Table 2:** DEMs in both the CG vs. TA2 and LTCG vs. LTTA2.

CG vs. TA2 and LTCG vs. LTTA2 Comparison	CG vs. TA2 log2FC	CG vs. TA2 Regulated	LTCG vs. LTTA2 log2FC	LTCG vs. LTTA2 Regulated
9α-(3-Methyl-2E-pentenoyloxy)-4S-hydroxy-10(14)-oplopen-3-one	−0.50	down	0.42	up
3-Methyl-3-butenyl apiosyl-(1->6)-glucoside	−0.56	down	0.53	up
N1-Acetyl-tabtoxinine-beta-lactam	−1.06	down	1.09	up
Deoxycholic acid	−1.11	down	0.81	up
Verbasoside	−1.04	down	0.67	up
Hypogeic acid	0.87	up	−0.94	down
3b,15b,17a-Trihydroxy-pregnenone	−0.88	down	−0.51	down
Cephalosporin C	2.17	up	2.30	up
Grepafloxacin	3.70	up	5.75	up
7-Hexadecynoic acid	0.45	up	−0.24	down
(2S)-2-(Diamino-methylidene-amino)-3-phenylpropanoic acid	36.89	up	37.21	up
N-Hexamethylene N′,N″-diethylene thio-phosphoramide	2.49	up	2.47	up
VORTIOXETINE	3.79	up	7.62	up
N-Succinyl-L-glutamate 5-semialdehyde	4.40	up	6.36	up

## Data Availability

All data generated and analyzed during this study are included in the published article.

## Supplementary Materials

The following supporting information can be downloaded at: https://www.mdpi.com/article/10.3390/ani15040544/s1, Table S1: The qRT-PCR primer sequences for the Chinese soft-shelled turtles; Table S2: The top 30 differentially expressed metabolites in CG vs. TA2 comparison; Table S3: The top 30 differentially expressed metabolites in LTCG vs. LTTA2 comparison; Table S4: The top 30 differentially expressed metabolites in CG vs. LTCG comparison; Table S5: The top 30 differentially expressed metabolites in TA2 vs. LTTA2 comparison; Table S6: The top 20 enrichment pathways of differentially expressed metabolites in CG vs.TA2 comparison; Table S7: The top 20 enrichment pathways of differentially expressed metabolites in LTCG vs. LTTA2 comparison; Table S8: The top 20 enrichment pathways of differentially expressed metabolites in CG vs. LTCG comparison; Table S9: The top 20 enrichment pathways of differentially expressed metabolites in TA2 vs. LTTA2 comparison.

## References

[1] Ji L.Q., Chen C., Zhu J.X., Hong X.Y., Liu X.L., Wei C.Q., Zhu X.P., Li W. (2024). Integrated time-series biochemical, transcriptomic, and metabolomic analyses reveal key metabolites and signaling pathways in the liver of the Chinese soft-shelled turtle (*Pelodiscus sinensis*) against Aeromonas hydrophila infection. Front. Immunol..

[2] Wang D., Gao H.Q. (2024). China Fishery Statistical Yearbook.

[3] Calabrese S., Nilsen T.O., Kolarevic J., Ebbesson L.O.E., Pedrosa C., Fivelstad S., Hosfeld C., Stefansson S.O., Terjesen B.F., Takle H. (2017). Stocking density limits for post-smolt Atlantic salmon (*Salmo salar* L.) with emphasis on production performance and welfare. Aquaculture.

[4] Zhang Z.B., Chen B.J., Yuan L., Niu C.J. (2015). Acute cold stress improved the transcription of pro-inflammatory cytokines of Chinese soft-shelled turtle against *Aeromonas hydrophila*. Dev. Comp. Immunol..

[5] Ai X.Q., Lin R., Ali Z.S., Zhu Q.J., Ding L., Shi H.T., Hong M.L. (2024). Seasonal changes in hepatic lipid metabolism and apoptosis in Chinese soft-shelled turtle (*Pelodiscus sinensis*). Comp. Biochem. Physiol. Part C Toxicol. Pharmacol..

[6] L’Honoré T., Farcy E., Blondeau-Bidet E., Lorin-Nebel C. (2020). Inter-individual variability in freshwater tolerance is related to transcript level differences in gill and posterior kidney of European sea bass. Gene.

[7] García Beltrán J.M., Esteban M.Á. (2022). Nature-identical compounds as feed additives in aquaculture. Fish Shellfish Immunol..

[8] Durazzo A., Lucarini M., Souto E.B., Cicala C., Caiazzo E., Izzo A.A., Novellino E., Santini A. (2019). Polyphenols: A concise overview on the chemistry, occurrence, and human health. Phytother. Res..

[9] Choi J.H., Liu G.C., Goo D.Y., Wang J.Q., Bowker B., Zhuang H., Kim W.K. (2022). Effects of tannic acid supplementation on growth performance, gut health, and meat production and quality of broiler chickens raised in floor pens for 42 days. Front. Physiol..

[10] Besharati M., Maggiolino A., Palangi V., Kaya A., Jabbar M., Eseceli H., De Palo P., Lorenzo J.M. (2022). Tannin in Ruminant Nutrition: Review. Molecules.

[11] Yang M.Q., Jiang D.H., Zhang L.L., Lu L.M., Xu Y., Khan M.S., Jiang J.C. (2025). Dietary condensed tannin supplementation improves growth performance and feed utilization of juvenile Largemouth bass (*Micropterus salmoides*) through positively regulating serum lipids and intestinal health. Aquaculture.

[12] Yang X.J., Zhou Y.X., Yu T.T., Li K., Xu S.W. (2024). TAN (tannic acid) inhibits BPA-induced pyroptosis of L8824 (grass carp hepatocytes) by regulating PTEN/PI3K/AKT pathway. Fish Shellfish Immunol..

[13] Ebrahim R., Liang J.B., Jahromi M.F., Shokryazdan P., Ebrahimi M., Li Chen W., Goh Y.M. (2015). Effects of Tannic Acid on Performance and Fatty Acid Composition of Breast Muscle in Broiler Chickens Under Heat Stress. Ital. J. Anim. Sci..

[14] Zhang Z.F., Xu P.T., Liu C.G., Chen J., Ren B.B., Du E.C., Guo S.S., Li P., Li L.L., Ding B.Y. (2024). Effect of Tannic Acid on Antioxidant Function, Immunity, and Intestinal Barrier of Broilers Co-Infected with Coccidia and *Clostridium perfringens*. Animals.

[15] Trefts E., Gannon M., Wasserman D.H. (2017). The liver. Curr. Biol..

[16] Morris A.M., Calsbeek D.J., Eckel R.H. (2004). Lipid metabolism and nutrient partitioning strategies. Curr. Drug Targets CNS Neurol. Disord..

[17] Bouchama A., Knochel J.P. (2002). Heat stroke. N. Engl. J. Med..

[18] Bacchetta C., Ale A., Rossi A.S., Karakachoff M., Cazenave J. (2020). Effects of cold stress on juvenile *Piaractus mesopotamicus* and the mitigation by β-carotene. J. Therm. Biol..

[19] Luo M.K., Feng B.B., Zhu W.B., Liang Z.Y., Xu W., Fu J.J., Miao L.H., Dong Z. (2024). Proteomics and metabolomics analysis of American shad (*Alosa sapidissima*) liver responses to heat stress. Comp. Biochem. Physiol. A Mol Integr. Physiol..

[20] Niu J.X., Wang Q.J., Jing C.W., Liu Y., Liu H., Jiao N., Huang L.B., Jiang S.Z., Guan Q.L., Li Y. (2022). Dietary *Galla Chinensis* tannic acid supplementation in the diets improves growth performance, immune function and liver health status of broiler chicken. Front. Vet. Sci..

[21] Houghton S.G., Cockerill F.R. (2006). Real-time PCR: Overview and applications. Surgery.

[22] Chen B., Qiu J.Q., Wang Y.X., Huang W., Zhao H.X., Zhu X.F., Peng K. (2022). Condensed tannins increased intestinal permeability of Chinese seabass (*Lateolabrax maculatus*) based on microbiome-metabolomics analysis. Aquaculture.

[23] Zhu X.F., Guo H., Li G.L., Zhu C.H. (2021). Effects of dietary hydrolyzable tannins on growth performance, antioxidant capacity, intestinal microflora and resistance against *Vibrio parahaemolyticus* of juvenile Pacific white shrimp, *Litopenaeus vannamei* (Boone, 1931). Aquac. Rep..

[24] Novriadi R., Hasan O.D.S., Nguyen K., Davies S., Panjaitan Z.G., Sektiana S.P., Gaddipati G.R., Trullàs C. (2023). Functional Effects of Hydrolyzable Tannins on the Growth, Health Status, and Hepatopancreas Histology of Pacific White Shrimp *Penaeus vannamei* Reared under Commercial Pond Conditions. Aquac. Res..

[25] Peng K., Zhou Y.H., Wang Y.X., Wang G.X., Huang Y.H., Cao J.M. (2020). Inclusion of condensed tannins in *Lateolabrax japonicus* diets: Effects on growth, nutrient digestibility, antioxidant and immune capacity and copper sulphate stress resistance. Aquac. Rep..

[26] Peng K., Chen B., Zhao H.X., Wang Y.X., Huang W. (2022). Condensed Tannins Improve Glycolipid Metabolism but Induce Liver Injury of Chinese Seabass (*Lateolabrax maculatus*). Front. Mar. Sci..

[27] Nawab A., Tang S.Y., Wen G., Guanghui L., Mei X., Lilong A., Jiang W., Wenchao L. (2024). Tannin Supplementation in Animal Feeding; Mitigation Strategies to Overcome the Toxic Effects of Tannins on Animal Health: A Review. J. Agric. Sci..

[28] Frutos P., Hervás G., Giráldez F.J., Mantecón A.R. (2004). Review. Tannins and ruminant nutrition. Span. J. Agric. Res..

[29] Guitart R., Croubels S., Caloni F., Sachana M., Davanzo F., Vandenbroucke V., Berny P. (2010). Animal poisoning in Europe. Part 1: Farm livestock and poultry. Vet. J..

[30] Tlak Gajger I., Dar S.A. (2021). Plant Allelochemicals as Sources of Insecticides. Insects.

[31] Zhang P., Liu N.C., Xue M.Y., Xiao Z.D., Zhang M.J., Meng Y., Fan Y.D., Hu X.W., Qiu J.Q., Zhang Q.H. (2023). Pathological characteristics of Chinese soft-shelled turtle (*Pelodiscus sinensis*) with white abdominal disease. Aquac. Rep..

[32] Martínez-Álvarez R.M., Morales A.E., Sanz A. (2005). Antioxidant Defenses in Fish: Biotic and Abiotic Factors. Rev. Fish Biol. Fish..

[33] Ighodaro O.M., Akinloye O.A. (2018). First line defence antioxidants-superoxide dismutase (SOD), catalase (CAT) and glutathione peroxidase (GPX): Their fundamental role in the entire antioxidant defence grid. Alex. J. Med..

[34] Wang Y.J., Chen Y., Zhang X.Y., Lu Y.P., Chen H.X. (2020). New insights in intestinal oxidative stress damage and the health intervention effects of nutrients: A review. J. Funct. Foods.

[35] Moine L., Rivoira M., Díaz de Barboza G., Pérez A., Tolosa de Talamoni N. (2018). Glutathione depleting drugs, antioxidants and intestinal calcium absorption. World J. Gastroenterol..

[36] Casas-Grajales S., Muriel P. (2015). Antioxidants in liver health. World J. Gastrointest. Pharmacol. Ther..

[37] Allameh A., Niayesh-Mehr R., Aliarab A., Sebastiani G., Pantopoulos K. (2023). Oxidative Stress in Liver Pathophysiology and Disease. Antioxidants.

[38] Chamorro S., Viveros A., Centeno C., Romero C., Arija I., Brenes A. (2013). Effects of dietary grape seed extract on growth performance, amino acid digestibility and plasma lipids and mineral content in broiler chicks. Animal.

[39] Vogeser M., Jacob K., Zachoval R. (2000). Erythrocyte protoporphyrins in hepatitis C viral infection. Clin. Biochem..

[40] Afonso S., Vanore G., Batlle A. (1999). Protoporphyrin IX and oxidative stress. Free Radic. Res..

[41] Sánchez-Marzo N., Lozano-Sánchez J., Cádiz-Gurrea M.L., Herranz-López M., Micol V., Segura-Carretero A. (2019). Relationships Between Chemical Structure and Antioxidant Activity of Isolated Phytocompounds from Lemon Verbena. Antioxidants.

[42] Jing W., Chunhua M., Shumin W. (2015). Effects of acteoside on lipopolysaccharide-induced inflammation in acute lung injury via regulation of NF-κB pathway in vivo and in vitro. Toxicol. Appl. Pharmacol..

[43] Zhou X.H., Zhang A.H., Wang L., Tan Y.L., Guan Y., Han Y., Sun H., Wang X.J. (2016). Novel chinmedomics strategy for discovering effective constituents from ShenQiWan acting on ShenYangXu syndrome. Chin. J. Nat. Med..

[44] Ozougwu J.C. (2017). Physiology of the liver. Int. J. Res. Pharm. Biosci..

[45] Jiao S., Nie M., Song H., Xu D., You F. (2020). Physiological responses to cold and starvation stresses in the liver of yellow drum (*Nibea albiflora*) revealed by LC-MS metabolomics. Sci. Total Environ..

[46] Tilg H., Moschen A.R. (2006). Adipocytokines: Mediators linking adipose tissue, inflammation and immunity. Nat. Rev. Immunol..

[47] Hardie D.G. (2014). AMPK--sensing energy while talking to other signaling pathways. Cell Metab..

[48] Yu M.H., Fan R.Y., Yang S.M. (2024). Effect of tannic acid on adiponectin and gonads in male Brandt’s voles (*Lasiopodomys brandtii*). Gen. Comp. Endocrinol..

[49] Zeng B.Y., Su M.H., Chen Q.X., Chang Q., Wang W., Li H.H. (2020). Anoectochilus roxburghii polysaccharide prevents carbon tetrachloride-induced liver injury in mice by metabolomic analysis. J. Chromatogr. B Analyt. Technol. Biomed. Life Sci..

[50] Cheng X.Y., Hu Y., Kuang J., Guo X.Q., Cao H.B., Wu H.S., Hu G.L., Zhuang Y. (2024). Berberine alleviates high-energy and low-protein diet-induced fatty liver hemorrhagic syndrome in laying hens: Insights from microbiome and metabolomics. Poult. Sci..

[51] Leaver M.J., Villeneuve L.A., Obach A., Jensen L., Bron J.E., Tocher D.R., Taggart J.B. (2008). Functional genomics reveals increases in cholesterol biosynthetic genes and highly unsaturated fatty acid biosynthesis after dietary substitution of fish oil with vegetable oils in Atlantic salmon (*Salmo salar*). BMC Genom..

[52] Paulusma C.C., Lamers W.H., Broer S., van de Graaf S.F. (2022). Amino acid metabolism, transport and signalling in the liver revisited. Biochem. Pharmacol..

[53] Geller D.A., Chia S.H., Takahashi Y., Yagnik G.P., Tsoulfas G., Murase N. (2001). Protective role of the L-arginine-nitric oxide synthase pathway on preservation injury after rat liver transplantation. J. Parenter. Enter. Nutr..

[54] Obayashi Y., Arisaka H., Yoshida S., Mori M., Takahashi M. (2015). The protection mechanism of proline from D-galactosamine hepatitis involves the early activation of ROS-eliminating pathway in the liver. SpringerPlus.

[55] Caballero B. (2005). Encyclopedia of Human Nutrition.

[56] Zhang Y., Dong L., Yang X., Shi H., Zhang L. (2011). α-Linolenic acid prevents endoplasmic reticulum stress-mediated apoptosis of stearic acid lipotoxicity on primary rat hepatocytes. Lipids Health Dis..

[57] Glencross B.D., De Santis C., Bicskei B., Taggart J.B., Bron J.E., Betancor M.B., Tocher D.R. (2015). A comparative analysis of the response of the hepatic transcriptome to dietary docosahexaenoic acid in Atlantic salmon (*Salmo salar*) post-smolts. BMC Genom..

[58] Song H.Z., Wu T., Xu D.D., Chu Q., Lin D.B., Zheng X.D. (2016). Dietary sweet cherry anthocyanins attenuates diet-induced hepatic steatosis by improving hepatic lipid metabolism in mice. Nutrition.

[59] Tao Y., Chen Y., Ren J., Jiang S., Zhang S., Xu H., Li Y. (2024). Lipidomics and transcriptomics analysis revealed the role of the spleen of Nile tilapia (*Oreochromis niloticus*) in lipid metabolism. Aquaculture.

[60] Feng R., Zhang Z., Guan Y.Q. (2021). Physiological and transcriptional analysis of Chinese soft-shelled turtle (*Pelodiscus sinensis*) in response to acute nitrite stress. Aquat. Toxicol..

