# Supplementary material for: Dietary Tannic Acid Improves Hepatic Health and Capacity to Deal with Temperature Fluctuation in the Chinese Soft-Shelled Turtle (Pelodiscus sinensis)
Source: Animals (Basel). 2025 Feb 13;15(4):544. doi: 10.3390/ani15040544 (PMC11851950; doi:10.3390/ani15040544)
Supplement: Supplementary file 1 [file animals-15-00544-s001.zip › animals-3450590-supplementary.pdf]

Supplementary table

Table S1. The qRT-PCR primer sequences for the Chinese soft-shelled turtles

Gene symbol	Gene name	Forward primer (5'-3')	Reverse primer (5'-3')	NCBI Accession number	product length (bp)
elof1	elongation factor 1	ACTCGTCCAACTGACAAGCCTC	CACGGCGAACATCTTTCACAG	XM_006124748	200
sod1	superoxide dismutase 1	GGACCACAAGATCAAGAGA	TAACACCACAAGCCAGAC	XM_006126060	229
Sod2	superoxide dismutase 2	GGTGTCTCTTCAGCCTGCCCTA	GCCAGAGCCTTGAACACCAACT	NM_001317049	196
cat	catalase	CGATTCTCCACTGTTGC	AGGTGAGTCTGAGGGTTC	NM_001286934	194
Gpx3	glutathione peroxidase 3	TGGATTTTCCCCTTGGTC	TGAGTCCTCAGTAGGTGGC	NW_005871017	202
Gpx4	glutathione peroxidase 4	GGCTCGCTGAGAGAGGTTTA	GGGCTGCTCTTTCATCCAT	NW_005855649	188

Table S2. The top 30 differentially expressed metabolites in CG vs. TA2 comparison.

Metabolites name	log2FC	Pvalue	VIP	regulated
7-Hexadecynoic acid	0.45	0.0004	2.67	up
2-Acetamidoethylphosphonate	-0.81	0.0004	2.69	down
Protoporphyrin	0.68	0.0021	2.50	up
Verbasoside	-1.04	0.0027	2.49	down
N-Succinyl-L-glutamate 5-semialdehyde	4.40	0.0030	2.61	up
2-Furoate	0.60	0.0031	2.44	up
(±)12,13-DiHOME	0.73	0.0037	2.57	up
(1E,4S,5E,7R)-Germacra-1(10),5-dien-11-ol	1.17	0.0038	2.39	up
(R)-4-(2-(2-(2-Methylpyrrolidin-1-yl)ethyl)benzofuran-5-yl)benzonitrile	-1.10	0.0040	2.55	down
Norcocaine	-0.73	0.0041	2.41	down
Butyryl timolol	-1.32	0.0041	2.48	down
6-Amino-6-deoxyfutalosine	1.08	0.0042	2.44	up
(S)-8-Oxocitronellyl enol	1.00	0.0044	2.50	up

Cephalosporin C	2.17	0.0046	2.42	up
1-(8Z,11Z,14Z-eicosatrienoyl)-sn-glycero-3-phosphocholine	0.68	0.0047	2.29	up
5-(12,15-Heneicosadienyl)-1,3-benzenediol	0.62	0.0049	2.41	up
3b,15b,17a-Trihydroxy-pregnenone	-0.88	0.0051	2.46	down
Bis(2-ethylhexyl) phthalate	-0.66	0.0054	2.57	down
3-Methyl-3-butenyl apiosyl-(1->6)-glucoside	-0.56	0.0056	2.32	down
10-Hexadecynoic acid	0.38	0.0059	2.37	up
LysoPC(20:0/0:0)	-1.19	0.0062	2.45	down
N-Hexamethylene N',N''-diethylene thiophosphoramide	2.49	0.0064	2.50	up
Cilengitide	1.24	0.0066	2.39	up
(-)-threo-Iso(homo)2-citrate	1.58	0.0066	2.32	up
N-Palmitoyl Arginine	-1.63	0.0073	2.50	down
Butanoyl PAF	-1.42	0.0075	2.42	down
((2-Amino-3-((2-amino-3-((carboxymethyl)amino)-3-oxopropyl)dithio)propanoyl)amino)acetic acid	0.68	0.0078	2.34	up
Cephaloridine	-1.10	0.0084	2.27	down
(5E)-5-[(3Ar,4S,5S,6aR)-5-hydroxy-4-[(E,3R)-3-hydroxyoct-1-enyl]-3,3a,4,5,6,6a-hexahydro-1H-pentalen-2-ylidene]pentanoic acid	-0.85	0.0086	2.34	down
PE(20:1(11Z)/PGJ2)	0.75	0.0087	2.45	up

Table S3. The top 30 differentially expressed metabolites in LTCG vs. LTTA2 comparison.

Metabolites name	log2FC	Pvalue	VIP	regulated
3-Methyl-3-butenyl apiosyl-(1->6)-glucoside	0.53	0.0016	2.77	up
4-Keto-anhydrotetracycline	-0.50	0.0021	2.72	down
5-HETE	0.61	0.0022	2.77	up
Heptadecanal	0.42	0.0034	2.83	up
N-Succinyl-L-glutamate 5-semialdehyde	6.36	0.0034	2.90	up
Menoctone	-1.21	0.0035	2.73	down

Anhydroamarouciaxanthin B	0.79	0.0044	2.67	up
2-Octaprenyl-6-hydroxyphenol	1.74	0.0056	2.59	up
LysoPC(18:4(6Z,9Z,12Z,15Z)/0:0)	-1.23	0.0060	2.68	down
(4Z,7Z,10E,12E,16Z)-18-(3-Ethylcycloprop-1-en-1-yl)-14-hydroxyoctadeca-4,7,10,12,16-pentaenoylcarnitine	-0.52	0.0066	2.67	down
Angiotensin (1-7)	0.89	0.0068	2.64	up
PG(16:0/18:2(9Z,12Z))	0.60	0.0069	2.65	up
DG(14:0/PGE2/0:0)	-1.36	0.0070	2.60	down
Deacetylisoipecoside	0.83	0.0076	2.65	up
Cephalosporin C	2.30	0.0082	2.44	up
10-OPDA	0.57	0.0082	2.53	up
9alpha-(3-Methyl-2E-pentenoyloxy)-4S-hydroxy-10(14)-oplopen-3-one	0.42	0.0082	2.57	up
C14:5n-1,3,5,7,9	0.49	0.0083	2.50	up
Cortisol 21-mesylate	0.81	0.0088	2.57	up
Geranylhydroquinone	0.52	0.0090	2.50	up
3,4-Dimethyl-5-pentyl-2-furanpropanoic acid	0.50	0.0092	2.49	up
Tanacetol B	0.61	0.0092	2.52	up
2-hexadecenal	0.45	0.0094	2.56	up
C20915	-1.33	0.0102	2.37	down
1,2,10-Trihydroxydihydro-trans-linalyl oxide 7-O-beta-D-glucopyranoside	0.64	0.0108	2.56	up
Pregna-1,9(11)-diene-3,20-dione, 21-(4-(2,6-di-1-pyrrolidinyl-4-pyrimidinyl)-1-piperazinyl)-16-methyl-, (5beta,16alpha)-	0.48	0.0112	2.47	up
Valienone 7-phosphate	1.01	0.0121	2.47	up
Dephospho-CoA	-0.67	0.0121	2.45	down
Reduced FMN	0.74	0.0123	2.48	up
Pregnanetriolone	1.37	0.0125	2.43	up

Table S4. The top 30 differentially expressed metabolites in CG vs. LTCG comparison.

Metabolites name	log2FC	Pvalue	VIP	regulated
4-Methylaminobutyrate	-1.65	0.0001	2.77	down
4-Keto-anhydrotetracycline	0.63	0.0003	2.70	up
Leucinic acid	30.79	0.0009	2.84	up
3-Methyl-3-butenyl apiosyl-(1->6)-glucoside	-0.58	0.0017	2.59	down
Arbutin 6-phosphate	0.62	0.0018	2.60	up
Cinchophen	0.39	0.0020	2.53	up
1,2,10-Trihydroxydihydro-trans-linalyl oxide 7-O-beta-D-glucopyranoside	-0.96	0.0023	2.59	down
Graveoline	0.40	0.0024	2.50	up
beta1-Chaconine	0.97	0.0026	2.43	up
3-Hydroxyadipic acid 3,6-lactone	0.43	0.0027	2.53	up
N-Acetyl-L-glutamate	-1.69	0.0037	2.60	down
(Hydroxymethylphenyl)succinyl-CoA	0.60	0.0039	2.48	up
Edulitine	0.42	0.0039	2.44	up
Indoxyl sulfate	-2.35	0.0042	2.44	down
N1-Acetyl-tabtoxinine-beta-lactam	-1.40	0.0044	2.35	down
Cefadroxil	0.38	0.0044	2.42	up
Cholic acid glucuronide	0.98	0.0054	2.38	up
3beta-Hydroxy-5alpha-pregnan-20-one acetate	0.83	0.0055	2.46	up
1-Methyl-2-undecylquinolin-4(1H)-one	-0.86	0.0055	2.51	down
Ceanothine D	-0.76	0.0055	2.40	down
Carbazochrome	-2.02	0.0061	2.29	down
1,4-Dihydro-2-methylbenzoicacid	-0.92	0.0065	2.28	down
Ampeloside Bf1	0.67	0.0066	2.53	up
CDP-DG(5-iso PGF2VI/a-21:0)	0.64	0.0072	2.45	up
Soyasaponin beta-A	0.65	0.0074	2.48	up
O-propanoyl-carnitine	-0.45	0.0075	2.23	down
PE(14:1(9Z)/22:6(5Z,8E,10Z,13Z,15E,19Z)-2OH(7S, 17S))	-1.31	0.0077	2.40	down
Calcidiol	1.48	0.0079	2.49	up

16-Glucuronide-estriol	-5.60	0.0093	2.57	down
O-Arachidonoyl Glycidol	0.81	0.0093	2.30	up

Table S5. The top 30 differentially expressed metabolites in TA2 vs. LTTA2 comparison.

Metabolites name	log2FC	Pvalue	VIP	regulated
7-Hexadecynoic acid	-0.48	0.0004	2.51	down
9alpha-(3-Methyl-2E-pentenoyloxy)-4S-hydroxy-10(14)-oplopen-3-one	0.54	0.0005	2.49	up
PG(16:0/18:2(9Z,12Z))	0.68	0.0006	2.52	up
2-Methylcitric acid	-0.81	0.0006	2.47	down
Avocadenofuran	-28.25	0.0007	2.54	down
N-Acetyl-L-glutamate	-1.27	0.0008	2.37	down
Deoxycholic acid	1.22	0.0010	2.49	up
L-Lactic acid	-0.82	0.0011	2.44	down
N-Palmitoyl Arginine	1.46	0.0013	2.57	up
Cephaloridine	1.20	0.0014	2.50	up
Pregna-1,9(11)-diene-3,20-dione, 21-(4-(2,6-di-1-pyrrolidinyl-4-pyrimidinyl)-1-piperazinyl)-16-methyl-, (5beta,16alpha)-	0.51	0.0014	2.41	up
6-Amino-6-deoxyfutalosine	-1.10	0.0016	2.41	down
L-Malic acid	-0.63	0.0019	2.51	down
(1E,4S,5E,7R)-Germacra-1(10),5-dien-11-ol	-1.35	0.0019	2.26	down
Naringenin	-0.76	0.0024	2.40	down
12,15-epoxy-13,14-dimethyleicosa-12,14-dienoic acid	1.11	0.0026	2.51	up
5-Hydroxy-L-tryptophan	-0.95	0.0027	2.48	down
(5E)-5-[(3Ar,4S,5S,6aR)-5-hydroxy-4-[(E,3R)-3-hydroxyoct-1-enyl]-3,3a,4,5,6,6a-hexahydro-1H-pentalen-2-ylidene]pentanoic acid	0.87	0.0028	2.21	up
Allopregnanolone	1.01	0.0036	2.40	up
PE(18:1(11Z)/16:0)	1.41	0.0037	2.44	up

R-1 Methanandamide	-0.70	0.0039	2.42	down
Reduced FMN	0.73	0.0040	2.37	up
Maleic acid	-0.71	0.0041	2.48	down
Butyryl timolol	1.20	0.0041	2.45	up
1-(8Z,11Z,14Z-eicosatrienoyl)-sn-glycero-3-phosphocholine	-0.70	0.0044	2.42	down
Verbasoside	0.98	0.0045	2.30	up
Vomifoliol	0.77	0.0046	2.49	up
Indoxyl sulfate	-2.42	0.0046	2.13	down
L-4-Hydroxy-3-methoxy-a-methylphenylalanine	-1.30	0.0051	2.23	down

Table S6. The top 20 enrichment pathways of differentially expressed metabolites in CG vs.TA2 comparison.

Pathway name	Differentially expressed metabolites (DEMs)	Count of DEMs	*P* value	enrichment factor
Bile secretion	Estradiol-17beta 3-glucuronide; Deoxycholic acid; Cephaloridine; Cefazolin	4	0.16	1.85
Steroid hormone biosynthesis	11beta,21-Dihydroxy-3,20-oxo-5beta-pregnan-18-al; 19-Hydroxytestosterone; Allopregnanolone; 3beta-Hydroxypregn-5-en-20-one sulfate; Estradiol-17beta 3-glucuronide	5	0.06	2.32
Pyruvate metabolism	Malonyl-CoA	1	0.34	2.49
AMPK signaling pathway	Malonyl-CoA	1	0.26	3.42
Drug metabolism - cytochrome P450	5-Phenyl-1,3-oxazinane-2,4-dione	1	0.65	0.98
Adipocytokine signaling pathway	Malonyl-CoA	1	0.04	27.36
Fatty acid biosynthesis	Malonyl-CoA	1	0.36	2.28
alpha-Linolenic acid metabolism	9(S)-HPOT	1	0.62	1.05
Caffeine metabolism	Xanthosine	1	0.23	3.91
Linoleic acid metabolism	12,13-Epoxy-9-hydroxy-10-octadecenoate	1	0.61	1.09

Antifolate resistance	AG 2034	1	0.36	2.28
Riboflavin metabolism	D-Ribulose 5-phosphate	1	0.43	1.82
Monobactam biosynthesis	N1-Acetyl-tabtoxinine-beta-lactam	1	0.55	1.30
Penicillin and cephalosporin biosynthesis	Cephalosporin C	1	0.31	2.74
Tyrosine metabolism	5,6-Indolequinone-2-carboxylic acid	1	0.51	1.44
Insulin resistance	Malonyl-CoA	1	0.14	6.84
Phenylalanine metabolism	2-Hydroxy-6-ketononatrienedioate; cis-3-(3-Carboxyethenyl)-3,5-cyclohexadiene-1,2-diol	2	0.14	3.04
ABC transporters	Methyl beta-D-galactoside; Xanthosine	2	0.63	0.96
Porphyrin metabolism	Protoporphyrin; L-Threonine O-3-phosphate	2	0.36	1.61
Drug metabolism - other enzymes	5'-Deoxy-5-fluorocytidine; 5-Fluorodeoxyuridine triphosphate	2	0.22	2.28
Nicotinate and nicotinamide metabolism	(S)-6-Hydroxynicotine	1	0.70	0.85
Phenylalanine, tyrosine and tryptophan biosynthesis	Shikimate 3-phosphate	1	0.49	1.52
Histidine metabolism	S-(Hercyn-2-yl)-L-cysteine S-oxide	1	0.61	1.09
beta-Alanine metabolism	Malonyl-CoA	1	0.36	2.28
Purine metabolism	Adenine; Oxalureate; Xanthosine	3	0.18	2.05
Insect hormone biosynthesis	Juvenile hormone III	1	0.51	1.44
One carbon pool by folate	5,10-Methenyltetrahydrofolate	1	0.23	3.91
Thiamine metabolism	Thiamine acetic acid	1	0.36	2.28
Glucagon signaling pathway	Malonyl-CoA	1	0.39	2.10
Tryptophan metabolism	8-Methoxykynurenate; Indole-3-ethanol	2	0.38	1.52
Arginine and proline metabolism	N-Succinyl-L-glutamate 5-semialdehyde	1	0.66	0.94
Pyrimidine metabolism	CMP	1	0.73	0.78
Propanoate metabolism	Malonyl-CoA	1	0.34	2.49
Alcoholic liver disease	Malonyl-CoA	1	0.11	9.12
Pentose phosphate pathway	D-Ribulose 5-phosphate	1	0.34	2.49

Folate biosynthesis	Dihydrobiopterin	1	0.62	1.05
Arachidonic acid metabolism	9(S)-HETE	1	0.77	0.70
Fatty acid elongation	Malonyl-CoA	1	0.31	2.74

Table S7. The top 20 enrichment pathways of differentially expressed metabolites in LTCG vs. LTTA2 comparison.

Pathway name	Count of differentially expressed metabolites	metabolites	Pvalue	enrichment factor
Parathyroid hormone synthesis, secretion and action	1	Calcidiol	0.14	6.57
Lipoic acid metabolism	1	Octanoic acid	0.20	4.60
Linoleic acid metabolism	1	Linoleic acid	0.43	1.84
Ubiquinone and other terpenoid-quinone biosynthesis	1	Geranylhydroquinone	0.46	1.64
Steroid biosynthesis	2	Calcidiol; 4alpha-Methylzymosterol-4-carboxylate	0.12	3.28
Arginine and proline metabolism	3	Nopaline; N-Succinyl-L-glutamate 5-semialdehyde; gamma-Glutamyl-gamma-aminobutyraldehyde	0.02	4.75
Neomycin, kanamycin and gentamicin biosynthesis	1	Lividomycin B	0.69	0.88
Monobactam biosynthesis	2	C20915; N1-Acetyl-tabtoxinine-beta-lactam	0.07	4.38
Steroid hormone biosynthesis	1	21-Deoxycortisol	0.74	0.78
alpha-Linolenic acid metabolism	1	10-OPDA	0.44	1.77
ABC transporters	1	Nopaline	0.72	0.81
Retinol metabolism	1	all-trans-4-Hydroxyretinoic acid	0.23	3.83

Arachidonic acid metabolism	1	5-HETE	0.58	1.18
Pantothenate and CoA biosynthesis	1	Dephospho-CoA	0.27	3.28
Bile secretion	1	Deoxycholic acid	0.74	0.78
Tuberculosis	1	Calcidiol	0.08	11.49
Riboflavin metabolism	1	Reduced FMN	0.28	3.06
Fatty acid biosynthesis	2	Octanoic acid; Tetradecanoic acid	0.03	7.66
Amino sugar and nucleotide sugar metabolism	1	UDP-N-acetylmuramate	0.50	1.48
Penicillin and cephalosporin biosynthesis	1	Cephalosporin C	0.20	4.60
Biosynthesis of unsaturated fatty acids	1	Linoleic acid	0.52	1.39
Renin-angiotensin system	1	Angiotensin (1-7)	0.20	4.60
Inflammatory mediator regulation of TRP channels	1	5-HETE	0.30	2.87
Porphyrin metabolism	2	Adenosyl cobyrinate hexaamide; 12-Ethyl-8-propylbacteriochlorophyllide d	0.17	2.70
Chemical carcinogenesis - DNA adducts	1	N-Sulfonyloxy-PhIP	0.30	2.87
Coronavirus disease - COVID-19	1	Angiotensin (1-7)	0.06	15.32

Table S8. The top 20 enrichment pathways of differentially expressed metabolites in CG vs. LTCG comparison.

Pathway name	Count of differentially expressed metabolites	metabolites	Pvalue	enrichment factor

Pentose and glucuronate interconversions	1	D-Ribulose 5-phosphate	0.28	3.06
Pantothenate and CoA biosynthesis	2	Dephospho-CoA; Uracil	0.11	3.49
Arachidonic acid metabolism	2	9(S)-HETE; 5-HETE	0.48	1.25
Nicotinate and nicotinamide metabolism	3	4-Methylaminobutyrate; FADH2; N1-Methyl-2-pyridone-5-carboxamide	0.14	2.29
Riboflavin metabolism	3	2-Amino-5-formylamino-6-(5-phospho-D-ribosylamino)pyrimidin-4(3H)-one; FADH2; D-Ribulose 5-phosphate	0.02	4.89
Parathyroid hormone synthesis, secretion and action	1	Calcidiol	0.25	3.49
Adipocytokine signaling pathway	1	Malonyl-CoA	0.04	24.45
Amino sugar and nucleotide sugar metabolism	2	UDP-N-acetylmuramate; Undecaprenyl phosphate alpha-L-Ara4N	0.36	1.58
Histidine metabolism	1	L-Histidine	0.65	0.98
Tuberculosis	1	Calcidiol	0.15	6.11
Central carbon metabolism in cancer	1	L-Histidine	0.65	0.98
Drug metabolism - cytochrome P450	1	Codeine-6-glucuronide	0.69	0.87
Protein digestion and absorption	1	L-Histidine	0.55	1.29
Biotin metabolism	1	Biotin	0.34	2.44
Vitamin digestion and absorption	2	Biotin; Vitamin D3	0.24	2.13
Tryptophan metabolism	1	5-(2'-Carboxyethyl)-4,6-dihydroxypicolinate	0.78	0.68
Monobactam biosynthesis	1	N1-Acetyl-tabtoxinine-beta-lactam	0.59	1.16
ABC transporters	2	L-Histidine; Biotin	0.69	0.86
AMPK signaling pathway	1	Malonyl-CoA	0.28	3.06
Glycolysis / Gluconeogenesis	2	2-(alpha-Hydroxyethyl)thiamine diphosphate; Arbutin 6-phosphate	0.08	4.07
Alcoholic liver disease	1	Malonyl-CoA	0.12	8.15
Arginine biosynthesis	1	N-Acetyl-L-glutamate	0.31	2.72

Phosphonate and phosphinate metabolism	1	Rhizocticin C	0.59	1.16
Insulin resistance	1	Malonyl-CoA	0.15	6.11
Polyketide sugar unit biosynthesis	1	dTDP-3-N,N-dimethylamino-2,3,6-trideoxy-4-keto-D-glucose	0.55	1.29
D-Amino acid metabolism	3	N-Acetyl-L-glutamate; L-Histidine; 1-Pyrroline-4-hydroxy-2-carboxylate	0.04	3.86
Fatty acid biosynthesis	2	Dodecanoic acid; Malonyl-CoA	0.08	4.07
Insect hormone biosynthesis	3	26-Hydroxyecdysone; 3-Dehydroecdysone; Farnesoic acid	0.04	3.86
Fatty acid elongation	1	Malonyl-CoA	0.34	2.44
Pyruvate metabolism	2	Malonyl-CoA; 2-(alpha-Hydroxyethyl)thiamine diphosphate	0.07	4.44
Inflammatory mediator regulation of TRP channels	1	5-HETE	0.49	1.53
Pentose phosphate pathway	2	D-Erythrose 4-phosphate; D-Ribulose 5-phosphate	0.07	4.44
Glucagon signaling pathway	1	Malonyl-CoA	0.42	1.88
Retinol metabolism	1	all-trans-4-Oxoretinoic acid	0.40	2.04
Propanoate metabolism	1	Malonyl-CoA	0.37	2.22
Folate biosynthesis	3	Biopterin; Dihydrobiopterin; 7-Carboxy-7-carbaguanine	0.09	2.82
Ubiquinone and other terpenoid-quinone biosynthesis	1	delta-Tocotrienol	0.69	0.87
Neomycin, kanamycin and gentamicin biosynthesis	1	6'''-Deamino-6'''-oxoneomycin C	0.89	0.47
Aminoacyl-tRNA biosynthesis	1	L-Histidine	0.51	1.44
Glyoxylate and dicarboxylate metabolism	1	Oxalyl-CoA	0.53	1.36
beta-Alanine metabolism	3	Malonyl-CoA; L-Histidine; Uracil	0.01	6.11
Vitamin B6 metabolism	1	D-Erythrose 4-phosphate	0.34	2.44
Lipoic acid metabolism	1	2-(alpha-Hydroxyethyl)thiamine diphosphate	0.34	2.44
Thermogenesis	1	FADH2	0.22	4.07
Phenylalanine, tyrosine and tryptophan biosynthesis	1	D-Erythrose 4-phosphate	0.53	1.36

Porphyrin metabolism	2	5-Oxo-delta-bilirubin; Calcidiol	0.41	1.44
Steroid biosynthesis	2	Vitamin D3; Calcidiol	0.32	1.75
Pyrimidine metabolism	2	Uracil; CMP	0.42	1.40
Biosynthesis of unsaturated fatty acids	2	(4Z,7Z,10Z,13Z,16Z,19Z)-Docosahexaenoic acid; Icosadienoic acid	0.39	1.48
Steroid hormone biosynthesis	2	2-Methoxy-estradiol-17beta 3-glucuronide; 16-Glucuronide-estriol	0.71	0.83
Citrate cycle (TCA cycle)	1	2-(alpha-Hydroxyethyl)thiamine diphosphate	0.22	4.07
Rheumatoid arthritis	1	Vitamin D3	0.08	12.22
Penicillin and cephalosporin biosynthesis	1	Deacetylcephalosporin C	0.34	2.44
Antifolate resistance	1	BGC 945	0.40	2.04

Table S9. The top 20 enrichment pathways of differentially expressed metabolites in TA2 vs. LTTA2 comparison.

Pathway name	Count of differentially expressed metabolites	metabolites	Pvalue	enrichment factor
Tryptophan metabolism	3	Formyl-5-hydroxykynurenamine; 5-Hydroxy-L-tryptophan; 5-(3'-Carboxy-3'-oxopropenyl)-4,6-dihydroxypicolinate	0.24	1.77
Inflammatory mediator regulation of TRP channels	1	(15S)-15-Hydroxy-5,8,11-cis-13-trans-eicosatetraenoate	0.54	1.33
Purine metabolism	2	Xanthosine; Adenine	0.57	1.06
Taste transduction	1	L-Malic acid	0.41	1.93

Primary bile acid biosynthesis	1	7alpha-Hydroxy-3-oxo-4-cholestenoate	0.64	1.01
Glutathione metabolism	2	Dehydroascorbic acid; Homotrypanothione	0.21	2.36
Metabolism of xenobiotics by cytochrome P450	1	Naphthalene	0.73	0.79
Thiamine metabolism	1	4-Amino-5-hydroxymethyl-2-methylpyrimidine	0.44	1.77
Riboflavin metabolism	1	Reduced FMN	0.52	1.42
Steroid biosynthesis	4	4alpha-Methylzymosterol-4-carboxylate; 3beta-Hydroxy-4beta,14alpha-dimethyl-9beta,19-cyclo-5alpha-ergost-24(24(1))-en-4alpha-carboxylate; trans,trans-Farnesyl diphosphate; Calcidiol	0.04	3.04
Pyruvate metabolism	2	L-Lactic acid; L-Malic acid	0.09	3.87
Glyoxylate and dicarboxylate metabolism	1	L-Malic acid	0.58	1.18
One carbon pool by folate	1	5-Formiminotetrahydrofolate	0.29	3.04
alpha-Linolenic acid metabolism	5	Traumatin; 10-OPDA; 2(R)-HOT; Jasmonic acid; OPC4-CoA	0.01	4.09
Linoleic acid metabolism	1	12,13-DHOME	0.70	0.85
Tyrosine metabolism	1	Maleic acid	0.60	1.12
Glucagon signaling pathway	2	L-Malic acid; L-Lactic acid	0.12	3.27
Proximal tubule bicarbonate reclamation	1	L-Malic acid	0.18	5.32
cAMP signaling pathway	1	L-Lactic acid	0.32	2.66

Ubiquinone and other terpenoid-quinone biosynthesis	2	Geranylhydroquinone; 6-Amino-6-deoxyfutalosine	0.38	1.52
Steroid hormone biosynthesis	5	17alpha,20alpha-Dihydroxypregn-4-en-3-one; 19-Hydroxytestosterone; Allopregnanolone; Pregnanediol; Pregnenolone	0.14	1.80
Citrate cycle (TCA cycle)	1	L-Malic acid	0.25	3.55
ABC transporters	5	L-Histidine; Methyl beta-D-galactoside; Xanthosine; 4-Amino-5-hydroxymethyl-2-methylpyrimidine; Oleandomycin	0.12	1.87
Ferroptosis	1	Adrenic acid	0.54	1.33
Nicotinate and nicotinamide metabolism	2	L-Malic acid; 4-Methylaminobutyrate	0.45	1.33
Neomycin, kanamycin and gentamicin biosynthesis	1	6'-Oxo-G418	0.92	0.41
Tuberculosis	1	Calcidiol	0.18	5.32
Insect hormone biosynthesis	1	trans,trans-Farnesyl diphosphate	0.60	1.12
Caffeine metabolism	1	Xanthosine	0.29	3.04
Fructose and mannose metabolism	1	L-Lactic acid	0.35	2.36
Arachidonic acid metabolism	3	(15S)-15-Hydroxy-5,8,11-cis-13-trans-eicosatetraenoate; 20-HETE; 8,9-DHET	0.28	1.64
Protein digestion and absorption	1	L-Histidine	0.60	1.12
Ascorbate and aldarate metabolism	2	D-Galactaro-1,5-lactone; Dehydroascorbic acid	0.11	3.55

Parathyroid hormone synthesis, secretion and action	1	Calcidiol	0.29	3.04
Antifolate resistance	1	AG 2034	0.44	1.77
Glycolysis / Gluconeogenesis	1	L-Lactic acid	0.44	1.77
Biosynthesis of unsaturated fatty acids	2	Adrenic acid; Eicosapentaenoic Acid	0.47	1.29
Phenylalanine metabolism	1	cis-3-(3-Carboxyethenyl)-3,5-cyclohexadiene-1,2-diol	0.58	1.18
Cushing syndrome	1	Pregnenolone	0.29	3.04
Renal cell carcinoma	1	L-Malic acid	0.05	21.28
HIF-1 signaling pathway	1	L-Lactic acid	0.21	4.26
Pathways in cancer	1	L-Malic acid	0.44	1.77
Central carbon metabolism in cancer	3	L-Lactic acid; L-Malic acid; L-Histidine	0.11	2.55
Propanoate metabolism	1	L-Lactic acid	0.41	1.93
Cortisol synthesis and secretion	1	Pregnenolone	0.29	3.04
beta-Alanine metabolism	1	L-Histidine	0.44	1.77
D-Amino acid metabolism	2	L-Histidine; N-Acetyl-L-glutamate	0.22	2.24
Valine, leucine and isoleucine degradation	1	(R)-3-Amino-2-methylpropanoate	0.32	2.66
Bile secretion	4	Cefazolin; Cephaloridine; Glucuronosyletoposide; Deoxycholic acid	0.30	1.44
Aminoacyl-tRNA biosynthesis	1	L-Histidine	0.56	1.25

Terpenoid backbone biosynthesis	2	trans,trans-Farnesyl diphosphate; Farnesylcysteine	0.09	3.87
Vascular smooth muscle contraction	1	20-HETE	0.21	4.26
Ovarian steroidogenesis	1	Pregnenolone	0.56	1.25
Aldosterone synthesis and secretion	1	Pregnenolone	0.41	1.93
Axon regeneration	1	5-Hydroxy-L-tryptophan	0.18	5.32
Serotonergic synapse	2	8,9-DHET; 5-Hydroxy-L-tryptophan	0.33	1.70
Arginine and proline metabolism	1	gamma-Glutamyl-gamma-aminobutyraldehyde	0.76	0.73
Histidine metabolism	1	L-Histidine	0.70	0.85
Monobactam biosynthesis	1	C20911	0.64	1.01
Butanoate metabolism	1	Maleic acid	0.35	2.36
Pyrimidine metabolism	1	neg_909	0.82	0.61
Efferocytosis	1	L-Lactic acid	0.32	2.66
Arginine biosynthesis	1	N-Acetyl-L-glutamate	0.35	2.36
